# MdSINA2‐MdNAC104 Module Regulates Apple Alkaline Resistance by Affecting γ‐Aminobutyric Acid Synthesis and Transport

**DOI:** 10.1002/advs.202400930

**Published:** 2024-07-20

**Authors:** Yuxing Li, Xiaocheng Tian, Tanfang Liu, Yanjiao Shi, Yunhao Li, Hongtao Wang, Yinglian Cui, Shuaiyu Lu, Xiaoqing Gong, Ke Mao, Mingjun Li, Fengwang Ma, Cuiying Li

**Affiliations:** ^1^ State Key Laboratory for Crop Stress Resistance and High‐Efficiency Production/Shaanxi Key Laboratory of Apple College of Horticulture Northwest A&F University Yangling Shaanxi 712100 China

**Keywords:** alkaline stress, apple, GABA, synthesis and efflux

## Abstract

Soil alkalization is an adverse factor limiting plant growth and yield. As a signaling molecule and secondary metabolite, γ‐aminobutyric acid (GABA) responds rapidly to alkaline stress and enhances the alkaline resistance of plants. However, the molecular mechanisms by which the GABA pathway adapts to alkaline stress remain unclear. In this study, a transcription factor, *MdNAC104* is identified, from the transcriptome of the alkaline‐stressed roots of apple, which effectively reduces GABA levels and negatively regulates alkaline resistance. Nevertheless, applying exogenous GABA compensates the negative regulatory mechanism of overexpressed *MdNAC104* on alkaline resistance. Further research confirms that *MdNAC104* repressed the GABA biosynthetic gene *MdGAD1/3* and the GABA transporter gene *MdALMT13* by binding to their promoters. Here, *MdGAD1/3* actively regulates alkaline resistance by increasing GABA synthesis, while *MdALMT13* promotes GABA accumulation and efflux in roots, resulting in an improved resistance to alkaline stress. This subsequent assays reveal that MdSINA2 interacts with MdNAC104 and positively regulates root GABA content and alkaline resistance by ubiquitinating and degrading MdNAC104 via the 26S proteasome pathway. Thus, the study reveals the regulation of alkaline resistance and GABA homeostasis via the MdSINA2‐MdNAC104‐*MdGAD1*/*3*/*MdALMT13* module in apple. These findings provide novel insight into the molecular mechanisms of alkaline resistance in plants.

## Graphical abstract

The MdSINA2‐MdNAC104‐*MdGAD1/MdGAD3*/*MdALMT13* module plays a critical role in regulating GABA homeostasis and the alkaline stress response of apple. These findings provide novel insight into the molecular mechanisms of alkaline resistance in plants.

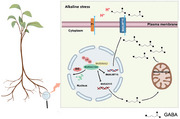

## 1 Introduction

Soil salinization affects more than 831 million hectares of land globally, seriously threatening plant growth and development.^[^ ^1^ ^]^ However, researchers have mostly focused on neutral salts such as sodium chloride (NaCl), but paid little attention to alkaline salts and high pH caused by sodium carbonate (Na_2_CO_3_) and sodium bicarbonate (NaHCO_3_). Soil alkalinity directly affects plant growth and soil fertility, especially the availability of nutrients and the production of harmful substances, which influence plant development. It is reported that 434 million hectares of soil in more than 100 countries suffer from soil alkalization; therefore, exploring the alkaline resistance genes and studying the underlying molecular mechanisms to improve alkaline resistance in plants is important.^[^ ^2^ ^]^

The newly discovered signal molecule, γ‐aminobutyric acid (GABA) plays a key role in plant resistance to abiotic stress.^[^ ^3^, ^4^ ^]^ Generally, when plants are subjected to unfavorable environmental factors such as heat, drought, and salinity, large amounts of GABA are accumulated in the plant temporarily. For example, under salt stress, GABA accumulates rapidly in plants, and the high amounts of GABA activate the H^+^‐ATPase, maintain a better membrane potential, reduce root K^+^ efflux, and lower cytoplasm Na^+^ levels.^[^ ^5^ ^]^ GABA also enhances plant resistance to heat and high light stress through autophagy.^[^ ^6^ ^]^ It also promotes stomatal closure and increases antioxidant capacity and photosynthesis to improve stress tolerance.^[^ ^3^, ^7^ ^]^ The synthesis of GABA occurs in the cytoplasm mainly through the action of glutamic acid decarboxylase (GAD).^[^ ^8^, ^9^ ^]^ In addition, aluminum‐activated malate transporters (ALMTs) have been reported to be directly acted upon by GABA and to facilitate GABA transport.^[^ ^10^, ^11^, ^12^, ^13^ ^]^ Therefore, maintaining higher levels of GABA content in plants to enhance resistance to adversity as an effective strategy demands manipulating these factors. Several studies have elucidated the GABA pathway in plants, primarily focusing on the exogenous processing and the pathway genes' function. However, studies exploring the regulatory mechanisms are rare.

The NAC family, named after the N‐terminally conserved NAC domains (*Petunia* NAM, *Arabidopsis* ATAF1/2, and CUC2),^[^ ^14^ ^]^ is a large family of transcription factors (TFs) unique to plants, including those involved in plant growth and development, metabolic processes, and adversity response. Numerous studies have reported the critical role of NAC transcription factors in defense against salt stress in different species. For instance, as the ectopic expression of sweet potato (*Ipomoea batatas*) *IbNAC3* enhanced salt resistance in *Arabidopsis thaliana*.^[^ ^15^ ^]^ Similarly, *BpNAC012* improved salt resistance in transgenic white birch (*Betula platyphylla*) by activating the expression of downstream genes encoding Δ−1‐pyrroline‐5‐carboxylate synthetase, superoxide dismutase, and peroxidase.^[^ ^16^ ^]^ However, there are only a few studies on NAC TFs in alkaline stress, especially the association with GABA.

Apples (*Malus domestica*) are one of the most economically valuable and nutritional fruit trees in the world. They are widespread woody perennials and grow well in soils with a pH of 5.5–6.7. However, soil alkalization in orchards severely restricts apple growth and yield. Interestingly, our previous study found that exogenous GABA improved apple resistance to alkaline stress, but its relationship with GABA synthesis and transport gene and the complex regulatory molecular mechanisms underlying this resistance remain unclear.^[^ ^17^ ^]^ In the present study, we identified an NAC transcription factor (MdNAC104) negatively correlated with the GABA content in roots. The overexpression of *MdNAC104* negatively regulated resistance to alkaline stress. Further, a series of biochemical assays showed that MdNAC104 was degraded by the E3 ubiquitin ligase MdSINA2 and directly inhibited the expression of the GABA biosynthetic gene *MdGAD1/3* and the transporter gene *MdALMT13*, thereby regulating GABA homeostasis and coordinating alkaline stress. These findings will reveal the GABA pathway‐associated regulatory network and provide a basis for breeding for alkaline resistance in plants.

## 2 Results

### 2.1 MdNAC104 Negatively Regulates Alkaline Resistance

**Figure 1:**
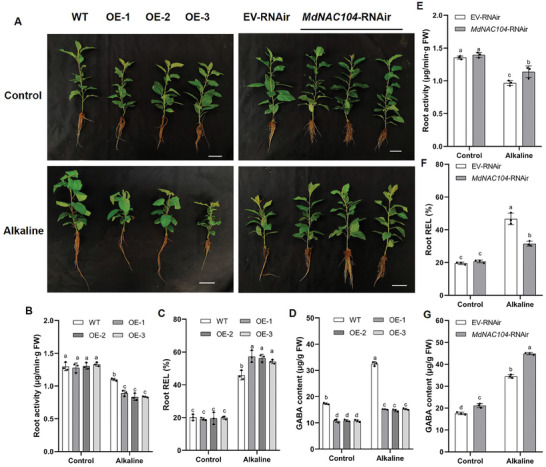
MdNAC104 reduces alkaline resistance in apple. A) Phenotypes of *MdNAC104*‐OE and *MdNAC104*‐RNAir (*MdNAC104*‐RNAi roots) treated for 20 days under hydroponic culture for alkaline stress (NaHCO_3_: Na_2_CO_3_ = 1:1), Bars = 5 cm. B,E) Root activity, C,F) Root relative electrolyte leakage (Root REL) and D,G) GABA content of transgenic *MdNAC104* apple. The data presented are mean ± standard deviation of three biological replicates. Different letters indicate significant differences in values as determined by a one‐way ANOVA Tukey”s test (*P* < 0.05).

We identified a transcription factor, *MdNAC104*, from the transcriptome of an already obtained alkaline‐stressed apple root.^[^ ^18^ ^]^ We found that the FPKM value of *MdNAC104* was significantly reduced under alkaline stress (Figure S1A, Supporting Information). RT‐qPCR detected reduced the expression of *MdNAC104* in roots under alkaline stress (Figure S1B, Supporting Information). Therefore, we utilized three previously obtained *MdNAC104* overexpression lines further to validate the gene's function under alkaline stress.^[^ ^19^, ^20^ ^]^ The growth of the *MdNAC104*‐OE apple plants was significantly inhibited after 20 days of alkaline stress (**Figure** **1**A). These plants demonstrated a significant reduction in plant height, biomass, and chlorophyll content and an increase in hydrogen peroxide (H_2_O_2_) content and superoxide anion (O_2_^−.^) content (Figure S2, Supporting Information). Roots are key receptors in response to alkaline stress, we measured the root activity and root REL (Relative electrolyte leakage). Further analysis revealed that *MdNAC104*‐OE lines had the lower root activity and higher root REL than the wild‐type (WT) plants under alkaline stress. However, no significant difference in root activity and root REL was observed between the OE and WT plants under normal conditions (Figure 1B,C). In addition, the *MdNAC104*‐RNAi roots (*MdNAC104*‐RNAir) generated using *Agrobacterium rhizogenes* showed the better growth than those with the empty vector (EV) under alkaline stress, and *MdNAC104*‐RNAir plants had the higher root activity and lower root REL (Figure 1A,E,F; Figure S3, Supporting Information). These observations suggest that MdNAC104 acts negatively under alkaline stress.

### 2.2 *MdNAC104* Alters GABA Content to Regulate Alkaline Resistance

Since GABA content is strongly correlated with alkaline stress,^[^ ^17^ ^]^ we found that the GABA content in the roots of *MdNAC104*‐OE lines was significantly lower than the WT under normal conditions, and this reduction was more pronounced under alkaline stress (Figure 1D). On the other hand, the *MdNAC104*‐RNAir plants showed an opposite trend (Figure 1G).

**Figure 2:**
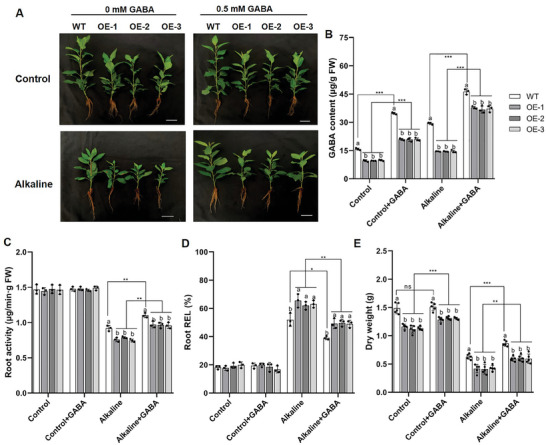
Exogenous GABA alleviates the phenotype of apple overexpressing *MdNAC104* under alkaline stress. A) Effects of 0.5 mm exogenous GABA on the growth of *MdNAC104*‐OE plants under alkaline stress for 20 days, Bars = 5 cm. B) GABA content, C) Root activity, and D) Root relative electrolyte leakage (Root REL) of transgenic *MdNAC104* apple. The data presented are mean ± standard deviation of three biological replicates. E) Dry weight. Data are mean ± standard deviation of five biological replicates. One‐way ANOVA Tukey's test (*P* < 0.05) and Student's *t*‐test were used to determine statistical significance (*P < 0.05, **P < 0.01, ***P < 0.001).

To further elucidate whether the reduced alkalinity resistance of *MdNAC104*‐OE is related to the decreased GABA content in roots, we treated the plants with 0.5 mM GABA under a hydroponic environment (**Figure** **2**A). The exogenous treatment with GABA increased the GABA content, plant height, and biomass of the *MdNAC104*‐OE under normal conditions (Figure 2B,E; Figure S4, Supporting Information). However, there were no significant differences in root activity and root REL between the *MdNAC104*‐OE and WT (Figure 2C,D). On the other hand, exogenous GABA alleviated the reduction of plant height, biomass, root activity, and the increase of root REL in both *MdNAC104*‐OE and WT lines under alkaline stress (Figure 2C–E; Figure S4, Supporting Information). Interestingly, we found that the features of *MdNAC104*‐OE lines (plant height, biomass, root activity, and root REL) under stress after exogenous GABA treatment were restored to those of the WT plants under stress without GABA (Figure 2A,C–E; Figure S4, Supporting Information). Besides, the exogenous GABA also alleviated the injury by alkaline stress in the WT plants. These results suggest that the negative role of MdNAC104 under alkaline stress is associated with the reduced GABA content in apple roots.

### 2.3 *MdNAC104* Directly Binds to the Promoters of *MdGAD1/3* and Inhibits Their Expression

**Figure 3:**
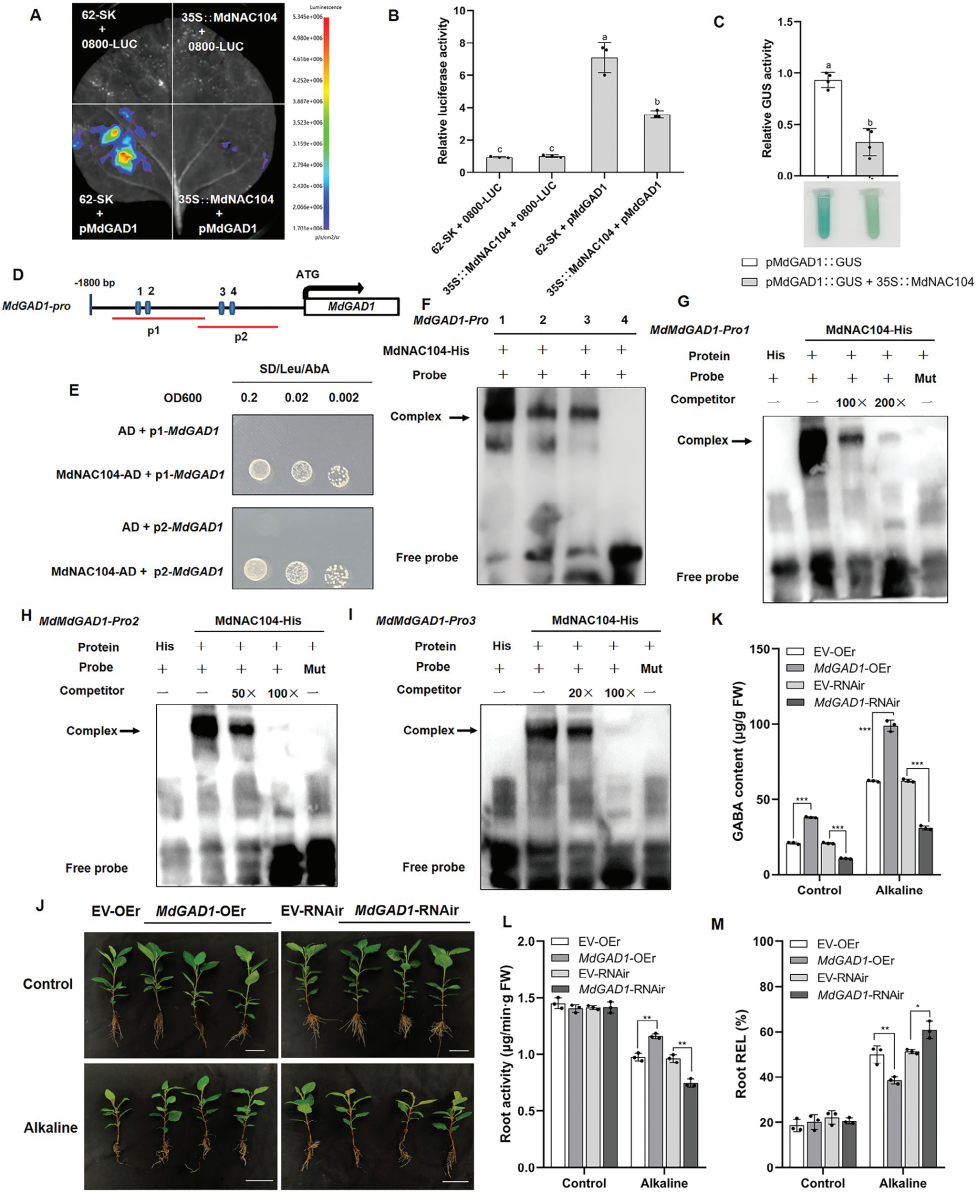
MdNAC104 inhibits the transcription of *MdGAD1* and binds to its promoter. A,B) Dual‐luciferase assay showing that MdNAC104 could inhibit the expression of *MdGAD1*. The relative luciferase (LUC:REN) activity of 62‐SK+0800‐LUC was set to 1. The effector vector (35S::MdNAC104) and the luciferase (LUC) reporter vector (pMdGAD1::LUC). Different letters indicate significant differences in values as determined by a one‐way ANOVA Tukey's test (*P* < 0.05). C) Relative β‐glucuronidase (GUS) activity. To observe the relationship between MdNAC104 and the *MdGAD1* promoter, *Agrobacterium tumefaciens* containing pMdGAD1::GUS + 35S (EV) and pMdGAD1::GUS + 35S::MdNAC104 were transformed into apple calli and stained. With pMdGAD1::GUS + 35S (EV) as the control, the activity of GUS was set as 1. Different letters indicate significant differences in values as determined by Student's *t*‐test (*P* < 0.05). D) “CACG” elements in the upstream promoter region of *MdGAD1*. E) Y1H assay. Yeast cotransformed with the empty pGADT7 (AD) vector and the *MdGAD1* promoter was used as a negative control. Yeast cotransformed with pAbAi‐p1‐*MdGAD1*/ pAbAi‐p2‐*MdGAD1* and MdNAC104‐AD transformants grew well on media supplemented with 60 and 100 ng mL^−1^ AbA, respectively. F–I) Identification of MdNAC104 binding specificity to sites 1, 2, and 3 in *MdGAD1*‐Pro by electrophoretic mobility shift assays. The “+” indicates the presence of relevant probes or proteins; “‐” indicates the absence of relevant proteins; “His” indicates pET‐32a vectors (His tag); Mut indicates the mutant form of *MdGAD1‐*pro in which the 5′‐CACG‐3′ has been replaced by 5′‐AAAA‐3′. The arrow indicates the position of a protein‐DNA complex after incubation of a biotin‐labelled DNA probe with His‐MdNAC104. J) Phenotypes of *MdGAD1*‐OEr (*MdGAD1*‐OE roots) and *MdGAD1*‐RNAir (*MdGAD1*‐RNAi roots) treated for 15 days under hydroponic culture for alkaline stress (NaHCO_3_: Na_2_CO_3_ = 1:1), Bars = 5 cm. K) GABA content, L) Root activity, and M) Root relative electrolyte leakage (Root REL) of transgenic *MdGAD1* apple. Data are mean ± standard deviation of three biological replicates. Student's *t*‐test were used to determine statistical significance (*P < 0.05, **P < 0.01, ***P < 0.001).

In higher plants, glutamic acid decarboxylase (GAD) is a key enzyme involved in the synthesis of GABA. Although the alkaline stress transcriptome showed little change in the FPMK values of the *MdGAD1* and *MdGAD3* genes at 6 h, 24 h, and 48 h compared to 0 h (Figure S5A,B, Supporting Information). RT‐qPCR results showed that the expression of *MdGAD1* was up‐regulated 1.8‐fold and 0.6‐fold after 3 h and 12 h of alkali stress treatment, respectively, compared with that at 0 h (Figure S5C, Supporting Information). The expression of *MdGAD3* was 3.1‐fold higher at 3 h than at 0 h, *MdGAD2* expression was not induced under alkaline stress (Figure S5C, Supporting Information). The expression levels of *MdGAD1* and *MdGAD3* were lower in *MdNAC104*‐OE than in the WT, but higher in *MdNAC104*‐RNAi roots than in the EV (Figure S5D,E, Supporting Information). We used a dual luciferase reporter gene system in tobacco to investigate the relationship between MdNAC104 and *MdGAD1* promoters. Here, the CDS sequence of *MdNAC104* was cloned into the effector vector (pGreenII62‐SK), and the promoter sequence of *MdGAD1* into the reporter gene vector (pGreenII0800‐Luc). Fluorescence was not detected when the two empty vectors were co‐expressed or when 0800‐LUC was co‐expressed with *MdNAC104* (**Figure** **3**A). However, when pMdGAD1 was co‐expressed with the empty effector vector, a clear fluorescent signal was observed, which was significantly suppressed by co‐expression of the MdNAC104 protein (Figure 3A). The fluorescence intensity observed was consistent with the relative LUC/REN activity (Figure 3B). Further, we used the β‐glucuronidase (GUS) reporter system in apple calli, and the GUS activity was significantly lower when pMdGAD1 was co‐expressed with 35S:MdNAC104 than when pMdGAD1 was co‐expressed with the EV (Figure 3C). This result was consistent with the dual luciferase activity results.

Subsequently, we performed a yeast one‐hybrid (Y1H) assays using the two fragments of *MdGAD1* promoter (p1 and p2) parts and revealed that MdNAC104 could bind to both p1 and p2 fragments (Figure 3D,E). Further, electrophoretic mobility shift assay (EMSA) was carried out to determine the sites where MdNAC104 binds to the *MdGAD1* promoter. The assay showed that MdNAC104 could directly bind to sites 1, 2, and 3 of the *MdGAD1* promoter, and mutation in these prevented binding (Figure 3F–I). Similar results were observed in assays that investigated bindings with *MdGAD3* (Figure S6, Supporting Information). These observations collectively suggested that MdNAC104 binds to the *MdGAD1/3* promoter and represses their expression.

### 2.4 MdGAD1/3 Positively Regulates Alkaline Resistance by Affecting GABA Content

To further validate the function of MdGAD1 under alkaline stress, we obtained transgenic apple roots (*MdGAD1*‐OEr, *MdGAD1*‐RNAir), apple calli, and heterologous overexpressed tomato (Figures S7 and S9A,E, Supporting Information). In this experiment, the transgenic apple roots (*MdGAD1*‐OEr, *MdGAD1*‐RNAir) were grown under alkaline stress for 15 days (Figure 3J). Under normal conditions, no significant difference was observed in plant height, fresh weight and dry weight of roots, root activity, and root REL in *MdGAD1*‐OEr, *MdGAD1*‐RNAir, and EV plants (Figure 3 L,M; Figure S8, Supporting Information). However, the content of GABA in *MdGAD1*‐OEr was higher than in the EV, while the content in *MdGAD1*‐RNAir was lower than in the EV (Figure 3K). Under alkaline stress, the plant height, fresh weight and dry weight of roots, root activity, and GABA content of *MdGAD1*‐OEr were higher than those of the EV, while the REL was lower than that of the EV (Figure 3K–M; Figure S8, Supporting Information). The *MdGAD1*‐RNAir plants showed an opposite trend (Figure 3J–M; Figure S8, Supporting Information). Subsequently, the transgenic apple calli were adopted to verify these results (Figure S9A, Supporting Information). Under alkaline stress, the fresh weight and GABA content of the calli overexpressing *MdGAD1* were significantly higher than in the WT, while the REL was lower than that of the WT (Figure S9B–D, Supporting Information). The heterologous expression of *MdGAD1* in tomato also resulted in similar changes (Figure S9E–K, Supporting Information). In addition, our experiments revealed that *MdGAD3* has the same impact as *MdGAD1* (Figure S10, Supporting Information). These results suggested that MdGAD1/3 positively regulates alkaline resistance by promoting the content of endogenous GABA.

### 2.5 *MdNAC104* Directly Binds to the Promoters of *MdALMT13* and Inhibits Its Expression

**Figure 4:**
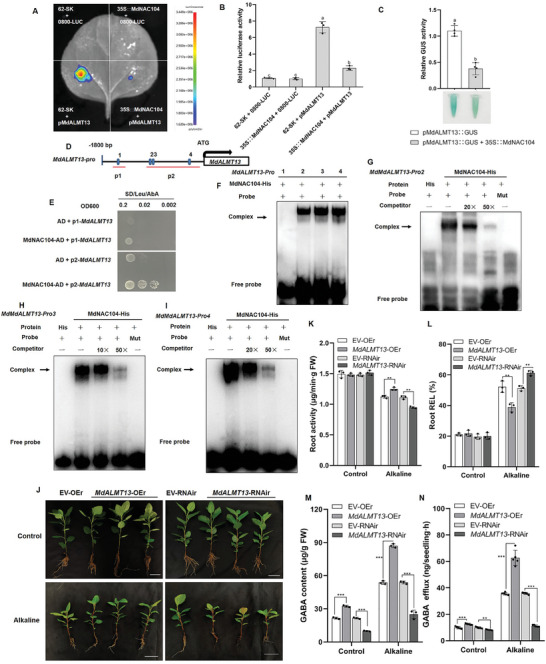
MdNAC104 directly suppresses *MdALMT13* transcription. A,B) Dual‐luciferase assay showing that MdNAC104 could inhibit the expression of *MdALMT13*. The relative luciferase (LUC:REN) activity of 62‐SK+0800‐LUC was set to 1. The effector vector (35S::MdNAC104) and the luciferase (LUC) reporter vector (pMdALMT13::LUC). Different letters indicate significant differences in values as determined by a one‐way ANOVA Tukey's test (*P* < 0.05). C) Relative β‐glucuronidase (GUS) activity. To observe the relationship between MdNAC104 and the *MdALMT13* promoter, *Agrobacterium tumefaciens* containing pMdALMT13::GUS + 35S (EV) and pMdALMT13::GUS + 35S::MdNAC104 were transformed into apple calli and stained. With pMdALMT13::GUS + 35S (EV) as control, the activity of GUS was set as 1. Different letters indicate significant differences in values as determined by Student's *t*‐test (*P* < 0.05). D) “CACG” elements in the upstream promoter region of *MdALMT13*. E) Y1H assay. Yeast cotransformed with the empty pGADT7 (AD) vector and the *MdALMT13* promoter was used as a negative control. Yeast cotransformed with pAbAi‐p3‐ *MdALMT13*/ pAbAi‐p4‐ *MdALMT13* and MdNAC104‐AD transformants grew well on media supplemented with 100 and 150 ng mL^−1^ AbA, respectively. F–I) Identification of MdNAC104 binding specificity to sites 2, 3, and 4 in *MdALMT13*‐Pro by electrophoretic mobility shift assays. The “+” indicates the presence of relevant probes or proteins; “‐” indicates the absence of relevant proteins; “His” indicates pET‐32a vectors (His tag); Mut indicates the mutant form of *MdALMT13*‐pro in which the 5′‐CACG‐3′ has been replaced by 5′‐AAAA‐3′. The arrow indicates the position of a protein‐DNA complex after incubation of a biotin‐labelled DNA probe with His‐MdNAC104. J) Phenotypes of *MdALMT13*‐OEr (*MdALMT13*‐OE roots) and *MdALMT13*‐RNAir (*MdALMT13*‐RNAi roots) treated for 15 days under hydroponic culture for alkaline stress (NaHCO_3_: Na_2_CO_3_ = 1:1), Bars = 5 cm. K) Root activity, L) Root relative electrolyte leakage (Root REL) and M) GABA content of transgenic *MdALMT13* apple. Data are mean ± standard deviation of three biological replicates. N) Net GABA efflux. The root of apple was placed in the corresponding solution, and the net GABA efflux for > 4 h was measured, and five biological repeats were set. Student's *t*‐test were used to determine statistical significance (*P < 0.05, **P < 0.01, ***P < 0.001).

ALMT is a GABA transporter. In this study, we identified a gene named *MdALMT13*, highly expressed in roots while responding to alkaline stress, using a transcriptome (Figure S11A, Supporting Information). RT‐qPCR showed that the expression of *MdALMT13* was up‐regulated under alkaline stress, increasing 11.6‐fold after 6 h (Figure S11B, Supporting Information). Interestingly, the expression of *MdALMT13* was lower in the *MdNAC104*‐OE line than in the WT, whereas it was higher in *MdNAC104*‐RNAir than in the EV control (Figure S11C,D, Supporting Information). Subcellular localization indicated that *MdALMT13* was localized to the cell membrane (Figure S12, Supporting Information). Further, dual luciferase and GUS activity analyses indicated that MdNAC104 repressed *MdALMT13* expression (**Figure** **4**A–C). Y1H assay and EMSA further determined that MdNAC104 could bind at sites 2, 3, and 4 of the *MdALMT13* promoter (Figure 4D–I). These results indicate that MdNAC104 directly binds to the promoter of *MdALMT13* and represses its expression.

### 2.6 MdALMT13 Positively Regulates Alkaline Resistance by Facilitating GABA Efflux

To verify whether MdALMT13 has GABA transporter ability, we used the 22Δ10α amino acid mutant and wild‐type 23344c strains. It was observed that both the 23344c, EV, and MdALMT13 grew normally on the medium with ammonium sulfate as the nitrogen source, while only MdALMT13 and 23344c grew on the medium with GABA as the nitrogen source (Figure S13, Supporting Information). To further investigate the MdALMT13 function, we obtained transgenic apple roots (*MdALMT13*‐OEr and *MdALMT13*‐RNAir, EV) with no significant difference in plant height, fresh weight and dry weight of roots, root activity, and root REL under normal conditions (Figure 4J–L; Figures S14 and S15, Supporting Information). Under alkaline stress, *MdALMT13*‐OEr had the higher plant height, fresh weight, and dry weight of roots, and root activity, and lower REL than the EV, while *MdALMT13*‐RNAir had the lower plant height, fresh weight, and dry weight of roots, and root activity, and higher root REL than the EV control (Figure 4K‐L; Figure S15, Supporting Information). Notably, we found that *MdALMT13*‐OEr secreted and accumulated more GABA in the roots than the EV, whereas *MdALMT13*‐RNAir had lower levels than the EV (Figure 4M,N). This pattern was more pronounced in plants under alkaline stress (Figure 4M,N). In addition, heterologous overexpression of *MdALMT13* in tomato resulted in the same trend (Figure S16, Supporting Information). We further found that the fresh weight and GABA content of *MdALMT13* overexpressing apple calli were significantly higher than those of the WT under alkaline stress, while the REL was significantly lower than that of WT calli (Figure S17, Supporting Information). Besides, the higher expression levels of *MdGAD1* and *MdGAD3* were detected in the transgenic *MdALMT13*‐OE apple calli than in the WT (Figure S18, Supporting Information). These results indicated that MdALMT13 positively regulates alkaline resistance to promote GABA efflux.

### 2.7 MdNAC104 Interacts with MdSINA2 to Promote MdNAC104 Degradation Through the 26s Proteasome Pathway

**Figure 5:**
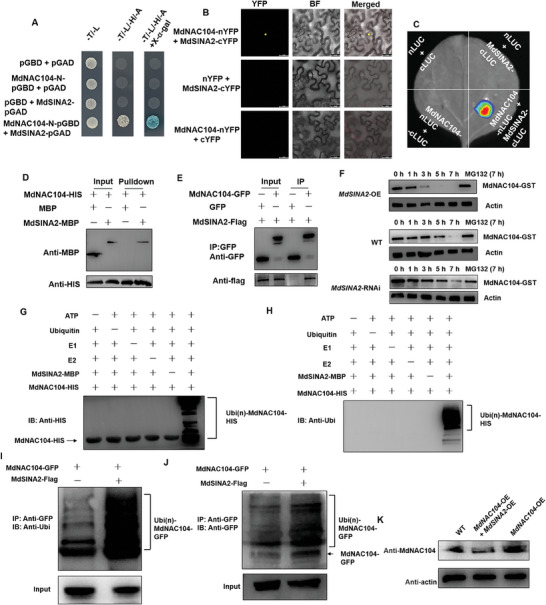
MdNAC104 interacts with MdSINA2 to promote MdNAC104 degradation through the 26s proteasome pathway. A) Interaction between MdNAC104 and MdSINA2 revealed by Y2H assays. MdSINA2‐pGAD and MdNAC104‐N‐pGBD was coexpressed in yeast and selected on SD/‐Trp‐Leu‐His‐Ade medium (‐T/‐L/‐H/‐A). B) BiFC assay. The fusion protein MdNAC104‐nYFP and MdSINA2‐cYFP were transiently co‐expressed in tobacco leaves and the fluorescence signal was observed. Bars = 30 µm. C) Split‐LUC assays. The fusion proteins MdNAC104‐nLuc and MdSINA2‐cLuc were transiently co‐expressed in tobacco leaves and the fluorescence signal was observed 48–60 h later using a plant live imaging system (PlantView100). D) Pull‐down assay. MdNAC104‐His protein was first incubated with anti‐His magnetic beads, followed by incubation with MBP or MdSINA2‐MBP. The bound proteins were eluted and detected with anti‐MBP and anti‐HIS antibodies. E) In vivo Co‐immunoprecipitation assay. The fusion proteins MdNAC104‐GFP and MdSINA2‐Flag were transiently co‐expressed in tobacco leaves. Total proteins were extracted and immunoprecipitated with anti‐GFP magnetic beads. Immunoblotting was performed using anti‐GFP and anti‐Flag antibodies. F) Cell‐free degradation assays of MdNAC104‐GST protein. Protein extracts from WT, *MdSINA2*‐OEr, and *MdSINA2*‐RNAir transgenic apple roots were incubated with MdNAC104‐GST protein for the indicated time periods. MdNAC104‐GST protein levels were detected by immunoblotting using anti‐GST antibody. For MG132 treatment, MG132 was added to the three protein extracts for 1 h followed by incubating with MdNAC104‐GTS protein for 7 h. ACTIN was used as an internal reference. G,H) Ubiquitination assays in vitro. The E3 ubiquitin ligase activity of MdSINA2‐MBP was tested in the presence and absence of ATP, ubiquitin, E1, E2, MdSINA2‐MBP, and MdNAC104‐HIS. The mixture was used to perform immunoblotting assays with anti‐HIS (G) and anti‐Ubi (H) antibodies. I,J) Ubiquitination analysis in vivo. I) Immunoblotting assays with anti‐ubiquitin (Ubi) antibody, J) Immunoblotting assays with anti‐GFP antibody. K) Protein abundance detection assay. MdNAC104 protein in transgenic apple roots overexpressing *MdNAC104* or co‐overexpressing *MdSINA2* and *MdNAC104* was detected using anti‐MdNAC104 antibody. Actin was used as an internal reference.

The abundance of MdNAC104 protein decreased in response to alkaline treatment (Figure S1C, Supporting Information). Therefore, we speculated that it might be degraded by ubiquitination modification. To verify this speculation, we used yeast two‐hybrid (Y2H) screening to identify an E3 ubiquitin ligase, MdSINA2, which was upregulated under alkaline stress (**Figure** **5**A; Figure S19, Supporting Information). Bimolecular fluorescence complementarity (BIFC) further verified the interaction between MdNAC104 and MdSINA2 in vivo (Figure 5B). Subsequently, the split‐luciferase complementation (Split‐LUC) assay confirmed that MdNAC104 interacted with MdSINA2. We fused MdNAC104 to the N‐terminal fragment of photinus luciferase (nLUC) and combined MdSINA2 with cLUC to obtain the fusion protein. In tobacco leaves co‐expressing MdNAC104‐nLUC and MdSINA2‐cLUC, recombinant LUC fluorescence was observed (Figure 5C). Then, pull‐down assay was performed to confirm this interaction in vitro (Figure 5D). Finally, the immunoprecipitation (Co‐IP) was performed to extract the proteins from tobacco leaves co‐expressing MdMdNAC104‐GFP and MdSINA2‐Flag. In this assay, MdNAC104‐GFP immunoprecipitated MdSINA2‐Flag (Figure 5E). Taken together, these in vivo and in vitro experiments indicated that MdNAC104 physically interacts with MdSINA2.

We further verified whether MdSINA2 mediated the degradation of MdNAC104. First, we incubated the total proteins of transgenic apple roots (*MdSINA2*‐OEr, WT, and *MdSINA2*‐RNAir) with MdNAC104‐GST fusion proteins for different periods (Figure S20A,B, Supporting Information). The degradation of MdNAC104 in *MdSINA2*‐OEr extracts increased with incubation time and its protein degradation was more rapid compared to the WT (Figure 5F). Meanwhile, the degradation of the MdNAC104 protein was significantly delayed in the *MdSINA2*‐RNAir extracts (Figure 5F). However, the presence of the proteasome inhibitor MG132 abolished the degradation of MdNAC104 (Figure 5F). Subsequently, the ubiquitination assay in vitro showed that MdSINA2 ubiquitinates MdNAC104 (Figure 5G,H). Meanwhile, the co‐expression of MdSINA2 and MdNAC104 resulted in a higher ubiquitination than only overexpression of MdNAC104 alone in vivo (Figure 5I,J). To test whether MdSINA2 controls MdNAC104 turnover, we examined the protein abundance of MdNAC104 in the transgenic apple roots. The analysis revealed a lower accumulation of MdNAC104 protein in roots overexpressing both MdSINA2 and MdNAC104 than in those overexpressing only MdNAC104 (Figure 5K). These results suggested that MdSINA2 mediates the ubiquitination and degradation of MdNAC104 via the 26S proteasome pathway.

### 2.8 MdSINA2 Regulates GABA Content and Apple Alkaline Resistance

**Figure 6:**
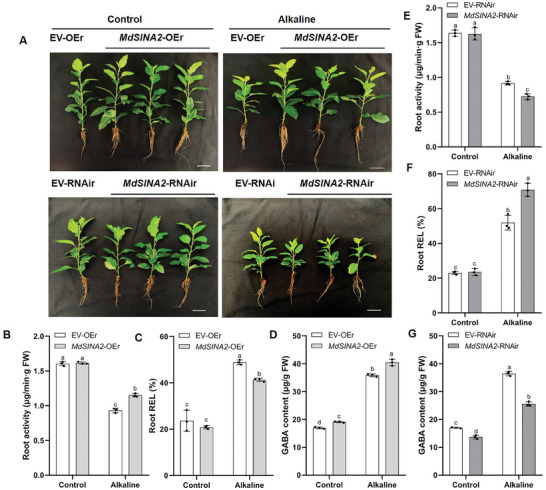
MdSINA2 positively regulates alkaline resistance in apples. A) *MdSINA2*‐OEr (*MdSINA2*‐OE roots) and *MdSINA2*‐RNAir (*MdSINA2*‐RNAi roots) phenotypes were subjected to alkaline stress (NaHCO_3_:Na_2_CO_3_ = 1:1) for 20 and 15 days, respectively. B,E) Root activity, C,F) Root relative electrolyte leakage (Root REL) and D,G) GABA content of transgenic MdSINA2 apple. The data presented are mean ± standard deviation of three biological replicates. Different letters indicate significant differences in values as determined by a one‐way ANOVA Tukey's test (*P* < 0.05).

We further detected upregulation of *MdSINA2* under alkaline stress based on the transcriptome data, so we generated transgenic apple roots of *MdSINA2* (*MdSINA2*‐OEr and *MdSINA2*‐RNAir) (**Figure** **6A**; Figure S20C,D, Supporting Information). The growth phenotype of transgenic apple roots under alkaline stress is shown in Figure 6A. Under alkaline stress, *MdSINA2*‐OEr plants showed the higher root activity, lower root REL, and significantly higher GABA content than the EV (Figure 6B–D). *MdSINA2*‐RNAir exhibited an opposite trend (Figure 6E–G). These results revealed that *MdSINA2* positively regulates the responses of apple to alkaline stress.

### 2.9 MdSINA2‐MdNAC104‐*MdGAD1/3*/*MdALMT13* Module Plays a Crucial Role in Maintaining GABA Homeostasis and Responding to Alkaline Stress

**Figure 7:**
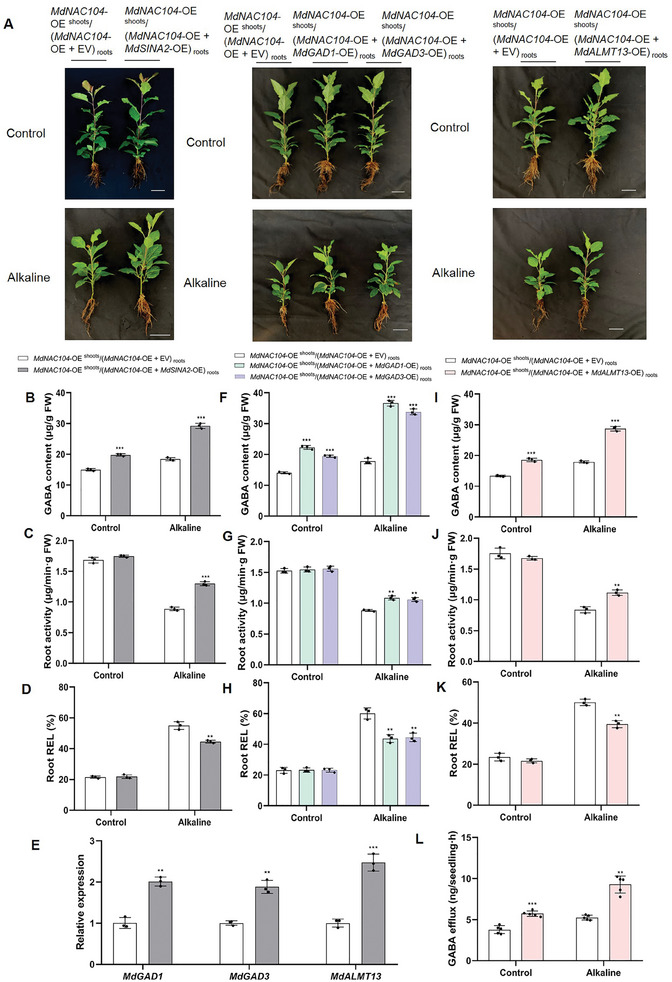
The MdSINA2‐MdNAC104‐*MdGAD1/3*/*MdALMT13* module plays a crucial role in maintaining GABA homeostasis and responding to alkaline stress. A) Chimeric plants were overexpressed for *MdNAC104‐*OE3 throughout the plant, while *MdSINA2*/*MdGAD1/3*/*MdALMT13* were overexpressed in hairy roots. Alkaline resistance of *MdNAC104*‐OE ^shoots^/(*MdNAC104*‐OE + *MdSINA2*‐OE) _roots_, *MdNAC104*‐OE ^shoots^/(*MdNAC104*‐OE + *MdGAD1*‐OE) _roots_, *MdNAC104*‐OE ^shoots^/(*MdNAC104*‐OE + *MdGAD3*‐OE) _roots_ and *MdNAC104*‐OE ^shoots^/(*MdNAC104*‐OE + *MdALMT13*‐OE) _roots_ transgenic apple plants in hydroponic culture. B,F,I) Root GABA content, C,G,J) Root activity, and D,H,K) Root relative electrolyte leakage (Root REL) of transgenic apple. E) The expression of *MdGAD1*, *MdGAD3*, and *MdALMT13* in roots cotransformed with *MdNAC104*‐OE3 and *MdSINA2*‐OE. The data presented are mean ± standard deviation of three biological replicates. L) Net GABA efflux. The root of apple was placed in the corresponding solution, and the net GABA efflux for > 4 h was measured, and five biological repeats were set. Student's *t*‐test were used to determine statistical significance (*P < 0.05, **P < 0.01, ***P < 0.001).

Based on the abovementioned results, we hypothesized that the MdSINA2‐MdNAC104‐*MdGAD1*/*3*/*MdALMT13* module played a key role in regulating GABA homeostasis and the response to alkaline stress. To verify this, we overexpressed *MdSINA2* (*MdNAC104*‐OE ^shoots^/(*MdNAC104*‐OE + *MdSINA2*‐OE) _roots_), *MdGAD1* (*MdNAC104*‐OE ^shoots^/(*MdNAC104*‐OE + *MdGAD1*‐OE) _roots_), *MdGAD3* (*MdNAC104*‐OE ^shoots^/(*MdNAC104*‐OE + *MdGAD3*‐OE) _roots_) and *MdALMT13* (*MdNAC104*‐OE ^shoots^/(*MdNAC104*‐OE + *MdALMT13*‐OE) _roots_) in the roots of *MdNAC104*‐OE3 overexpressing transgenic apple seedlings (**Figure** **7**A; Figure S21, Supporting Information). Analysis of these plants revealed that under normal conditions, *MdNAC104*‐OE ^shoots^/(*MdNAC104*‐OE + *MdSINA2*‐OE) _roots_ plants accumulated more GABA compared to *MdNAC104*‐OE ^shoots^/ (*MdNAC104*‐OE + EV) _roots_ plants (Figure 7B). Meanwhile, compared with the *MdNAC104*‐OE ^shoots^/ (*MdNAC104*‐OE + EV) _roots_ plants, *MdNAC104*‐OE ^shoots^ /(*MdNAC104*‐OE + *MdSINA2*‐OE) _roots_ plants had the higher GABA content and root activity, and lower root REL under alkaline stress (Figure 7B–D). Similar results were detected in *MdNAC104*‐OE ^shoots^/(*MdNAC104*‐OE + *MdGAD1*‐OE) _roots_ plants, *MdNAC104*‐OE ^shoots^/(*MdNAC104*‐OE + *MdGAD3*‐OE) _roots_ plants and *MdNAC104*‐OE ^shoots^/(*MdNAC104*‐OE + *MdALMT13*‐OE) _roots_ plants (Figure 7F–K). Interestingly, the transcriptional levels of *MdGAD1*, *MdGAD3* and *MdALMT13* in *MdNAC104*‐OE ^shoots^/(*MdNAC104*‐OE + *MdSINA2*‐OE) _roots_ plants were higher than *MdNAC104*‐OE ^shoots^/(*MdNAC104*‐OE + EV) _roots_ plants (Figure 7E). Under normal conditions, *MdNAC104*‐OE ^shoot^ (*MdNAC104*‐OE + *MdALMT13*‐OE) _roots_ plants secreted more GABA than *MdNAC104*‐OE ^shoots^/(*MdNAC104*‐OE + EV) _roots_ plants (Figure 7L). Besides, alkaline stress exacerbated this process (Figure 7L). These results confirmed that the MdSINA2‐MdNAC104‐*MdGAD1/3*/*MdALMT13* module is important in balancing GABA homeostasis and regulating alkaline stress in apple.

## 3 Discussion

Soil alkalization causes damage to plants, such as growth inhibition and metabolic disorders. Recently, a few genes related to alkaline stress have been reported in plants.^[^ ^13^, ^21^, ^22^ ^]^ However, these genes are less reported in apple, one of the perennial woody fruit trees. Thus, the mechanisms underlying alkaline stress also remained unclear. In this study, we identified a transcription factor, *MdNAC104*, from the transcriptome of alkaline‐stressed roots; this transcription factor was found to be downregulated under stress (Figure S1, Supporting Information).^[^ ^18^ ^]^ Moreover, overexpression of *MdNAC104* showed reduced root activity and increased REL under alkaline stress, while silencing in roots (*MdNAC104‐*RNAir) showed the opposite trend (Figure 1). Interestingly, we found that overexpression of *MdNAC104* altered the content of GABA and reduced the resistance to alkaline stress, while the exogenous GABA partially restored the growth phenotype and alkaline stress resistance of *MdNAC104*‐overexpressing plants (Figure 2). Previous studies have explained the function of NAC TFs in stress and metabolism.^[^ ^23^, ^24^ ^]^ In rice (*Oryza sativa* L.), OsNAC23 plays a role in regulating sugar content and yield.^[^ ^25^ ^]^ Besides, NAC42 is known to regulate rice (*Oryza sativa* L.) nitrogen utilization under low nitrogen conditions.^[^ ^26^ ^]^ In rose (*Rosa chinensis*), overexpression of *RcNAC091* improved drought resistance through an abscisic acid‐dependent pathway.^[^ ^27^ ^]^

Research has proven that GABA enhances plant resistance under alkaline stress.^[^ ^17^, ^28^ ^]^ In higher plants, GAD plays a key role in synthesizing GABA.^[^ ^29^ ^]^ Shelp et al. identified five GAD genes in *Arabidopsis thaliana*, and explored the functions of various *GADs*.^[^ ^30^ ^]^ Meanwhile, Mekonnen et al. found that the *Arabidopsis gad1/2* mutant was more sensitive to drought stress because of reduced GABA content and increased stomatal conductance.^[^ ^31^ ^]^ The *gad1* mutant also showed significant growth inhibition under phosphorus‐deficiency conditions compared with WT in *Arabidopsis*.^[^ ^32^ ^]^ In 2013, three *MdGADs* were identified in apple. Further Li et al. reported a positive effect of *MdGAD1* on Glomerella leaf spot (GLS).^[^ ^33^, ^34^ ^]^ Our study detected a significant reduction in GABA content in apple roots due to MdNAC104., Besides, lower transcript levels of *MdGAD1* and *MdGAD3* were detected in roots overexpressing *MdNAC104* compared to WT, while opposite was found in the RNAi plants (Figure S5D,E, Supporting Information). Y1H, LUC activity, GUS activity, and EMSA assays further verified that MdNAC104 could bind directly to the promoter of *MdGAD1* (Figure 3A–I). The *MdGAD1* transgenic calli, tomato plants and apple roots confirmed the positive role of this gene in regulating alkaline stress resistance by increasing GABA content (Figure 3J–M; Figures S8 and S9, Supporting Information). Similar results were observed in *MdGAD3‐*overexpressing apple roots (Figure S10, Supporting Information). Co‐expression of *MdGAD1*/*3* in *MdNAC104*‐overexpressing apple roots enhanced the resistance to alkaline stress (Figure 7). These results suggested that the MdNAC104‐*MdGAD1/3* module modulates alkaline resistance by influencing endogenous GABA synthesis.

In plants, ALMT family genes are involved in a range of functions, such as aluminum resistance, symbiotic nitrogen fixation, mineral nutrition, anion homeostasis, and fruit flavor.^[^ ^35^ ^]^ From the transcriptome of apple roots under alkaline stress, we identified 28 *MdALMT* genes and focused on a gene highly expressed in roots and highly responsive to alkaline stress, named *MdALMT13* (Figure S11, Supporting Information). The gene was found to be localized to the cell membrane (Figure S12, Supporting Information). Further genetic transformation showed that MdALMT13 positively regulates alkaline stress (Figure 4J–N; Figures S15–S17, Supporting Information). A few recent studies have indicated that ALMTs function as a transporter of GABA. Similarly, we found a MdALMT13 with GABA transporting ability using yeast mutants (Figure S13, Supporting Information). Also, *MdALMT13*‐overexpressing plants had more GABA content in the hydroponic solution than EV, while the opposite was observed with the RNAi plants (Figure 4N). Of course, researchers have reported that GABA efflux from the rhizosphere plays a positive role in regulating response under alkaline pH and in improving the soil microbial community.^[^ ^13^, ^36^ ^]^ Interestingly, the GABA content of *MdALMT13‐*overexpressing plants was significantly higher than the WT (Figure 4M; Figure S17D, Supporting Information). Further, we found that *MdGAD1/3* was transcribed at a higher level in *MdALMT13* transgenic calli than in WT (Figure S18, Supporting Information). Besides, the expression of *MdALMT13* in the *MdNAC104‐*overexpressing plants was lower than that in the WT, whereas the expression in the RNAi plant was higher than in the WT (Figure S11C,D, Supporting Information). A series of biochemical assays demonstrated that MdNAC104 could bind directly to the promoter of *MdALMT13* (Figure 4A–I). Moreover, the overexpression of *MdALMT13* in *MdNAC104*‐OE roots enhanced its resistance to alkaline stress, while endogenous GABA content and efflux were significantly increased (Figure 7). These observations suggested that the MdNAC104‐*MdALMT13* module played an important role in regulating GABA homeostasis and alkalinity resistance.

Ubiquitination is a post‐translational modification that selectively regulates the abundance and dynamic balance of cellular components in eukaryotes.^[^ ^37^, ^38^, ^39^ ^]^ In plants, this process helps cope with adversity. Generally, protein ubiquitination involves E1 ubiquitin‐activating enzymes, E2 ubiquitin‐binding enzymes, and E3 ubiquitin ligases. Among these components, E3 ubiquitin ligases are key enzymes in the ubiquitin pathway that specifically recognize target proteins. In particular, RING‐type ubiquitin ligases are actively involved in plant stress responses and are of numerous types.^[^ ^40^ ^]^ SINA5 (Seven in absentia), a RING‐type E3 ubiquitin ligase, was first identified in *Arabidopsis thaliana*.^[^ ^41^ ^]^ Subsequently, numerous SINAs were further reported.^[^ ^42^, ^43^, ^44^ ^]^ Here, we screened a *MdSINA2* gene by Y2H (Figure 5A). The transcriptome and RT‐qPCR results showed an induced expression of this ligase under alkaline stress, with the highest expression observed in roots compared to other *MdSINA* genes (Figure S19, Supporting Information). Further functional validation revealed a positive effect of MdSINA2 under alkaline stress (Figure 6). In *Arabidopsis*, AtSINA1‐4 have been reported to promote the degradation of FREE1 proteasome and positively regulate the effect of plants under iron deficiency.^[^ ^45^ ^]^ The SINA E3 ubiquitin ligase of rice (*Oryza sativa* L.), OsDIS1, negatively regulates drought stress resistance by degrading OsNek6.^[^ ^46^ ^]^ Our experiments demonstrated that MdNAC104 protein levels gradually decreased under alkaline stress, indicating that MdSINA2 promoted the ubiquitination and degradation of MdNAC104 (Figure 5F–K). Interestingly, with the overexpression of *MdSINA2* in the roots of *MdNAC104*‐OE plants, we found higher transcript levels of *MdGAD1/3* and *MdALMT13* than with the overexpression of *MdNAC104* only; enhanced GABA content and alkalinity resistance were also detected in these plants (Figure 7).

**Figure 8:**
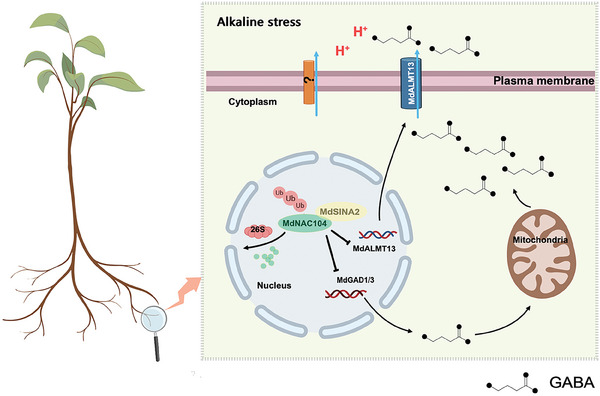
A model of the MdSINA2‐MdNAC104‐*MdGAD1/ MdGAD3/ MdALMT13* module regulating GABA homeostasis in roots and responding to plant alkaline resistance. First, MdNAC104 can directly bind to the promoters of the GABA synthase genes *MdGAD1* and *MdGAD3* to inhibit their transcription and reduce GABA accumulation in roots. Subsequently, MdNAC104 can also directly bind to the promoter of the GABA transporter gene *MdALMT13* to inhibit its transcription and reduce GABA accumulation and efflux in roots. Overexpression of *MdNAC104* decreased the expression of *MdGAD1, MdGAD3* and *MdALMT13* genes and lowered GABA levels in the roots induced by alkaline stress, thereby reducing the plant's alkaline resistance. In addition, alkaline stress inhibited *MdNAC104* expression and protein stability, thereby promoting MdSINA2‐mediated ubiquitination and MdNAC104 degradation. This process may have maintained root GABA homeostasis and alkalinity resistance in apple.

In summary, the present study identified a novel pathway based on SINA E3 ubiquitin ligase (MdSINA2)‐MdNAC104 that influences GABA synthesis and transport in apple roots under alkaline stress (**Figure** **8**). We found that MdNAC104 negatively regulates alkaline stress by repressing the GABA biosynthetic gene *MdGAD1/3*, and the ability of the GABA transporter gene *MdALMT13* to efflux GABA. We also found a gradual decrease in MdNAC104 protein level under alkaline stress. In apple, the E3 ubiquitin ligase MdSINA2 positively regulated alkaline stress. Finally, we found that MdSINA2 ubiquitinated and degraded MdNAC104 to affect GABA homeostasis. Importantly, this MdSINA2‐MdNAC104‐*MdGAD1/3*/*MdALMT13* module played a critical role in regulating GABA homeostasis and the alkaline stress response of apple.

## 4 Experimental Section

### Plant Materials and Alkaline Treatment

Overexpressed *MdNAC104* transgenic apple plants (OE#1, OE#2, and OE#3) were from Jia et al. and Mei et al.^[^ ^19^, ^20^ ^]^ “GL‐3′ (a variant of ‘Royal Gala”) and *Malus hupehensis* seedlings were used as materials for genetic transformation of transgenic apple roots. The methods for multiplication and rooting of ‘GL‐3′ were described by Guo et al.^[^ ^47^, ^48^ ^]^ After the apomixis seedlings of *M. hupehensis* were stratified in wet sand at 0 °C −4 °C for 40 days, the seeds with the same germination state were selected for sowing.^[^ ^17^ ^]^ When grew to 5–6 true leaves, the seedlings (‘GL‐3′ and *M. hupehensis*) with the same growth were selected for alkaline treatment. Through the hydroponic culturing techniques, alkaline stress was simulated according to the method of Li et al.^[^ ^17^, ^49^ ^]^ Briefly, the 1/2 Hoagland nutrient solution was adjusted to pH 9 (1 m NaHCO_3_: 1 m Na_2_CO_3_ = 1:1) and pH 6, and the nutrient solution was replaced every 5 days.^[^ ^50^ ^]^ The method of exogenous GABA was based on the previous study.^[^ ^17^ ^]^

The calli of WT and transgenic apple were cultured on the medium (4.43 g L^−1^ MS, 30 g L^−1^ sucrose, 1.5 mg L^−1^ 2,4‐D and 0.4 mg L^−1^ 6‐BA) for 10 days, then the calli of the same weight were cultured in the dark on the above medium of pH = 9 (1 mgf NaHCO_3_: 1 m Na_2_CO_3_ = 1:1) and pH = 6, and the phenotype was observed.

Transgenic tomato plants were transplanted and cultured according to Zhu et al.^[^ ^51^ ^]^ Tomato seedlings with the uniform growth were selected for the hydroponic culture for 10 d, and then treated for alkaline stress with pH 9 nutrient solution, with pH 6 nutrient solution as the control.

### Stable Transformation of Apple Roots, Apple Calli and Tomato

The transformation of transgenic apple roots (‘GL‐3′ and *Malus hupehensis*) was conducted following a previously described.^[^ ^52^ ^]^ The pCambia2300‐MdSINA2/MdGAD1/MdGAD3/MdALMT13 (overexpression vector, GFP‐tag) and pK7GWIWG2D‐MdSINA2/MdNAC104/MdGAD1/MdALMT13 (RNAi vector, GFP‐tag) recombinant plasmid were transformed into K599 *Agrobacterium rhizogenes* and used for root infection ‘GL‐3′ and *M. hupehensis*. ‘GL‐3′ was used to generate MdSINA2 and MdNAC104 transgenic root. *M. hupehensis* was used to generate *MdGAD1*, *MdGAD3*, and *MdALMT13* transgenic root. The acquisition of MdNAC104‐OE^shoots^/(MdNAC104‐OE+MdGAD1‐OE)_roots_, MdNAC104‐OE^shoots^/(MdNAC104‐OE+MdGAD3‐OE)_roots_, MdNAC104‐OE^shoots^/(MdNAC104‐OE+MdALMT13‐OE)_roots_ and MdNAC104‐OE^shoots^/ (MdNAC104‐OE+MdSINA2‐OE)_roots_ transgenic, briefly, the recombinant plasmid (pCambia2300‐MdSINA2/MdGAD1/MdGAD3/MdALMT13) were transformed into K599 and then infiltrated into MdNAC104 overexpressing OE‐3 line transgenic apple.

The recombinant pCambia2300‐MdALMT13 plasmid was transformed into *Agrobacterium tumefaciens* EHA105 to infiltrate Orin apple calli.^[^ ^53^ ^]^ The pCambia2300‐MdGAD1 and pCambia2300‐MdALMT13 constructs were transformed into tomato (*Solanum lycopersicum* cv. Micro‐Tom), as described by Sun et al.^[^ ^54^ ^]^

### Measurement of the GABA Efflux, GABA Content, Root Relative Electrolyte Leakage (REL) and Root Activity

For the determination of GABA efflux from transgenic roots, in brief, apple seedlings and tomato seedlings with the same growth were selected to grow in 500 mL (per replicate) of aerated 1/2 Hoagland nutrient solution for 7–10 d, then the transgenic roots were cleaned and the corresponding pH 1/2 Hoagland nutrient solution (pH = 6 and pH = 9) was replaced for the treatment. Four seedlings were selected as one biological replicates, and five replicates were carried out. Each replicate treatment was not < 4 h, after which solution samples were collected for determination.

GABA was extracted and measured as described by LC‐MS (LC: AC, Exion LC; MS: Q‐Trap5500, AB Sciex Pret) according to the description of Li et al.^[^ ^34^ ^]^ Briefly, 0.1 g of fresh samples were homogenised with 1 mL of 50% ethanol solution (containing 0.1 mol L^−1^ hydrochloric acid), then centrifuged and filtered through a 0.22 µm filter and finally diluted tenfold with methanol (Sigma–Aldrich) and detected.

The method of measuring the REL according to Dionisio‐Sese and Tobita,^[^ ^55^ ^]^ using the Ray Magnetic DDS‐307 conductivity meter (Leici Instrument Co., Ltd., Shanghai, China). According to the manufacturer's instructions, the extraction and determination of apple root activity was carried out using a Comin Biotechnology kit (Suzhou, China).

### Yeast GABA Transport Assays

Yeast GABA transport assays were performed as previously described by Besnard et al. and Guo et al.^[^ ^56^, ^57^ ^]^ Briefly, the full‐length CDS sequence of the *MdALMT13* was ligated into pYES2 vector, transformed into the yeast amino acid transporter mutation 22Δ10α and wild‐type 23344c strains, and grown for 2 days in YNB solid medium with 1 mM (NH_4_)_2_SO_4_ as the nitrogen source, then switched to YNB solid medium with 1 mM GABA as the nitrogen source to observe growth.

### RNA Extraction and RT‐qPCR Analysis

Total RNA from different tissues of the plant was analyzed with the RNA extraction kit (Tiangen Biotech Co., Ltd). RNA was reverse transcribed into cDNA and analyzed by RT‐qPCR using a LightCycler 96 system (Roche, Switzerland) according to the manufacturer's protocol. The expression of related genes was calculated according to Livak and Schmittgen.^[^ ^58^ ^]^ The stably expressed *Malus* elongation factor 1 alpha gene (EF‐1**α**; DQ341381) was used as the reference gene. The primer sequences of *MdGADs* related genes refered to previous studies.^[^ ^33^, ^59^ ^]^ The primers are shown in Table S1 (Supporting Information).

### Protein Purification

The CDS region of MdNAC104 was connected to pET‐32a (His‐tag), and pGEX4T‐1 (GST‐tag) vectors, respectively, whereas the CDS region of MdSINA2 was connected to pMAL‐c5X (MBP‐tag) vectors. Protein purification was based on the study of Li et al.^[^ ^34^ ^]^ Purification of MdNAC104‐His, MdNAC104‐GST and MdSINA2‐MBP was performed according to the instructions of Ni‐NTA His·bind resin purification resin (7sea biotech. China) and ProteinIso GST/MBP Resin (TRAN, China).

### Transcription Factor–DNA Interaction Validation

For dual‐luciferase assay, the coding sequence *MdNAC104* was inserted into the effector vector pGreenII 62‐SK, and the promoters of *MdGAD1*/*3* and *MdALMT13* were inserted into the reporter vector pGreenII 0800‐Luc, respectively, and the corresponding vectors were transformed into GV3101 (p19), which was co‐infiltrated into the tobacco leaves. After 48–60 h, the fluorescence imaging was observed using the plant imaging system (PlantView100; Guangzhou Bio‐Optics Biotechnology Co., Ltd., China), and the luciferase activity was determined by dual luciferase reporter gene analysis kit (Shanghai Yisen Bio‐Optics Biotechnology Co., Ltd., China).

For Y1H assay, CDS of MdNAC104 was constructed into pGADT7 vector. The promoter sequences of *MdGAD1* and *MdALMT13* were constructed into the pAbAi vector and yeast cells were transformed. The lowest concentration of AbA (aureobasidin A), which inhibited yeast growth, was screened on SD/Ura medium. Yeast containing pAbAi‐proMdGAD1, pAbAi‐proMdGAD3 and pAbAi‐proMdALMT13 fusion expression vectors were transformed into receptor cells, and pGADT7‐MdNAC104 plasmid was transformed into the previously prepared receptor cells. Clones screened from SD/‐Ura‐Leu medium were inoculated into SD/‐Ura‐Leu medium containing AbA, and the growth of yeast was observed after 2 days.

For GUS assay, the promoters of *MdGAD1*/*3* and *MdALMT13* were connected to the pCAMBIA0390 vector containing the GUS reporter gene to construct the pMdGAD1/3::GUS and pMdALMT13::GUS reporter gene expression vectors. The CDS of MdNAC104 was inserted into the pCAMBIA2300 vector to construct the 35S::MdNAC104 expression vector as an effector vector. The recombinant plasmid was transformed into *Agrobacterium* tumefaciens GV3101 to infiltrate apple calli. The GUS staining and assay were performed according to the previous description.^[^ ^60^ ^]^

For EMSA, probes were synthesized by Shanghai Biotech Co., Ltd. and EMSA analysis was performed using the LightShift Chemiluminescent EMSA Kit according to the manufacturer's instructions (Thermo Fisher Scientific Inc., USA).

### Protein–Protein Interaction Validation

For Y2H assay, the vector of MdSINA2‐pGADT7 and MdNAC104‐N‐pGBKT7 (1‐132aa: contains NAC conserved domain) was co‐transformed into the yeast strain Y2H‐Gold. They were cultured on SD‐Trp‐Leu medium for 2 days, respectively, and then the clones screened in SD‐Trp‐Leu medium were inoculated into SD‐Ade‐His‐Trp‐Leu medium and imaged after 3 days.

For Split‐LUC assay, the CDS regions of MdNAC104 and MdSINA2 were connected to the vector containing nLUC and cLUC, respectively. The recombinant plasmid was transformed into *Agrobacterium tumefaciens* GV3101, and the imaging was observed with a plant live imaging system (PlantView100; Guangzhou Bio‐Optics Biotechnology Co., Ltd., China) at 48–60 h after the injection of post‐tobacco.

For BiFC assay, MdNAC104 and MdSINA2 full‐length genes were cloned into pSPYNE and pSPYCE vectors to construct MdNAC104‐nYFPN and MdSINA2‐cYFP, respectively. The recombinant plasmids were transformed into *Agrobacterium* tumefaciens C58C1 and co‐expressed in tobacco leaves. The fluorescence of yellow fluorescent protein (YFP) was detected after 48 h by laser scanning confocal microscopy (TCS SP8; Leica, Germany) to detect the fluorescence of YFP.

For pull‐down assay, the MdNAC104‐His and MdSINA2‐MBP recombinant proteins were obtained and the proteins were blotted with antibodies labeled with His and MBP according to the manufacturer's instructions using the Anti‐His magnetic beads (P2135; Beyotime Biotechnology Co., Ltd., Shanghai, China) method.

For Co‐IP assay, the MdNAC104‐GFP, MdSINA2‐FLAG, and GFP proteins were expressed in specific combinations in tobacco leaves. After 2 days of incubation, total proteins were extracted from the leaves and incubated with anti‐GFP magnetic beads (Beyotime Biotechnology Co., Ltd., Shanghai, China) overnight at 4 °C. Western blot was detected with anti‐FLAG and anti‐GFP antibodies.

### Ubiquitination Assay In Vitro and In Vivo

For the in vitro ubiquitination assay, ATP, E1, E2, E3 (MdSINA2‐MBP) and substrate proteins (MdNAC104‐His) were incubated simultaneously at 30 °C for 10 h. Western blot was detected with anti‐ His and anti‐Ubi antibodies.

Ubiquitination assay according to Liu et al. in vivo.^[^ ^61^ ^]^ Briefly, the constructed MdNAC104‐GFP, MdSINA2‐FLAG vector, transformed with *Agrobacterium tumefaciens* GV3101 was used to infest tobacco with specific combinations. The total protein was extracted after 2 days and detected by western blot with anti‐GFP antibody and anti‐Ubi antibody.

### Cell‐Free Protein Degradation Assay In Vitro

Total protein from transgenic apple roots (MdSINA2‐OE and MdSINA2‐RNAir) was extracted and added to MdNAC104‐GST purified proteins co‐incubated at 22 °C and sampled at different time points. At 0 h samples or treatment with proteasome inhibitor MG132 (100 µM) were used as the control. The protein level of MdNAC104‐GST was detected by GST antibody.

### Statistical Analysis

SPSS software (Version 22.0) was used for statistical analysis. The data were processed by a one‐way analysis of variance (ANOVA) for Tukey's test (*P* < 0.05) or Student's *t*‐test (*P < 0.05, **P < 0.01, ***P < 0.001). The value is expressed as mean ± standard deviation (SD).

### Accession Numbers

MdNAC104 (MF401514.1/MD15G1415700), MdSINA2 (MD01G1218700), MdGAD1 (NM_001294057.1/MD14G1242700), MdGAD3 (NM_001294084.1/ MD16G1010800), and MdALMT13 (XM_008388287.3/MD11G1287000).

## Conflict of Interest

The authors declare no conflict of interest.

## Author Contributions

Y.L. and X.T. contributed equally to this work. C.L. and Y.L. conceived and designed the experiments; Y.L. performed the experiments with assistance from X.T., T.L., Y.S., Y.L., H.W., C.L., and S.L.; Y.L. and X.T. analyzed the data; Y.L. wrote the paper; C.L. and F.M. provided financial support and helped to perform the analysis with constructive discussions; X.G. and K.M. provided assistance for materials; F.M. provided materials and laboratory apparatus. All the authors approved the final manuscript.

## Supporting information

Supporting Information

Supplemental Table 1

## Data Availability

The data that support the findings of this study are available on request from the corresponding author. The data are not publicly available due to privacy or ethical restrictions.
