# Supplementary material for: MdSINA2‐MdNAC104 Module Regulates Apple Alkaline Resistance by Affecting γ‐Aminobutyric Acid Synthesis and Transport
Source: Adv Sci (Weinh). 2024 Jul 20;11(35):2400930. doi: 10.1002/advs.202400930 (PMC11425205; doi:10.1002/advs.202400930)
Supplement: Supplementary file 1 — Supporting Information [file ADVS-11-2400930-s002.docx]

**MdSINA2-MdNAC104 module regulates apple alkaline** **resistance by affecting γ-aminobutyric acid synthesis and transport**

Yuxing Li^†^, Xiaocheng Tian^†^, Tanfang Liu, Yanjiao Shi, Yunhao Li, Hongtao Wang, Yinglian Cui, Shuaiyu Lu, Xiaoqing Gong, Ke Mao, Mingjun Li, Fengwang Ma*, Cuiying Li*

*State Key Laboratory for Crop Stress Resistance and High-Efficiency Production*/*Shaanxi Key Laboratory of Apple, College of Horticulture,* *Northwest A&F University, Yangling, Shaanxi 712100, China*

^*^Corresponding authors:

Cuiying Li

E-mail: lcy1262@nwafu.edu.cn

Fengwang Ma

E-mail: fwm64@sina.com, fwm64@nwsuaf.edu.cn

^†^These authors contributed equally to this work.


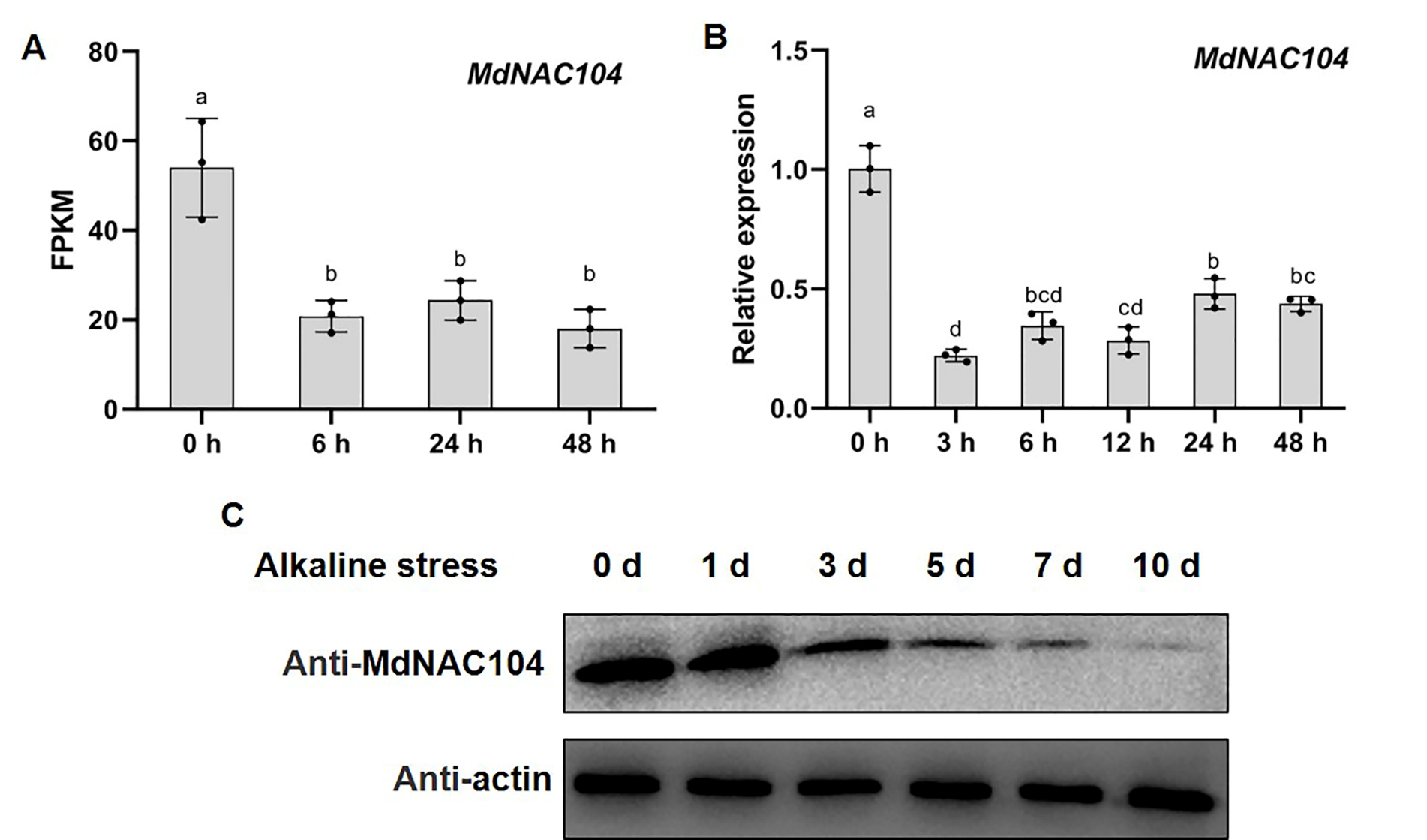


Figure S1. Effects of alkaline stress on *MdNAC104* expression and protein abundance in apple roots. (A) The FPKM of *MdNAC104* in apple roots under alkaline stress based on RNA-seq data. (B) RT-qPCR analysis of *MdNAC104* expression under alkaline stress. Data are mean ± standard deviation of three biological replicates. (C) Protein abundance of MdNAC104 in apple roots under alkaline stress. Immunoblotting was performed using anti-MdNAC104 antibodies. Actin was used as an internal reference. Different letters indicate significant differences in values as determined by a one-way ANOVA Tukey’s test (*P* < 0.05).


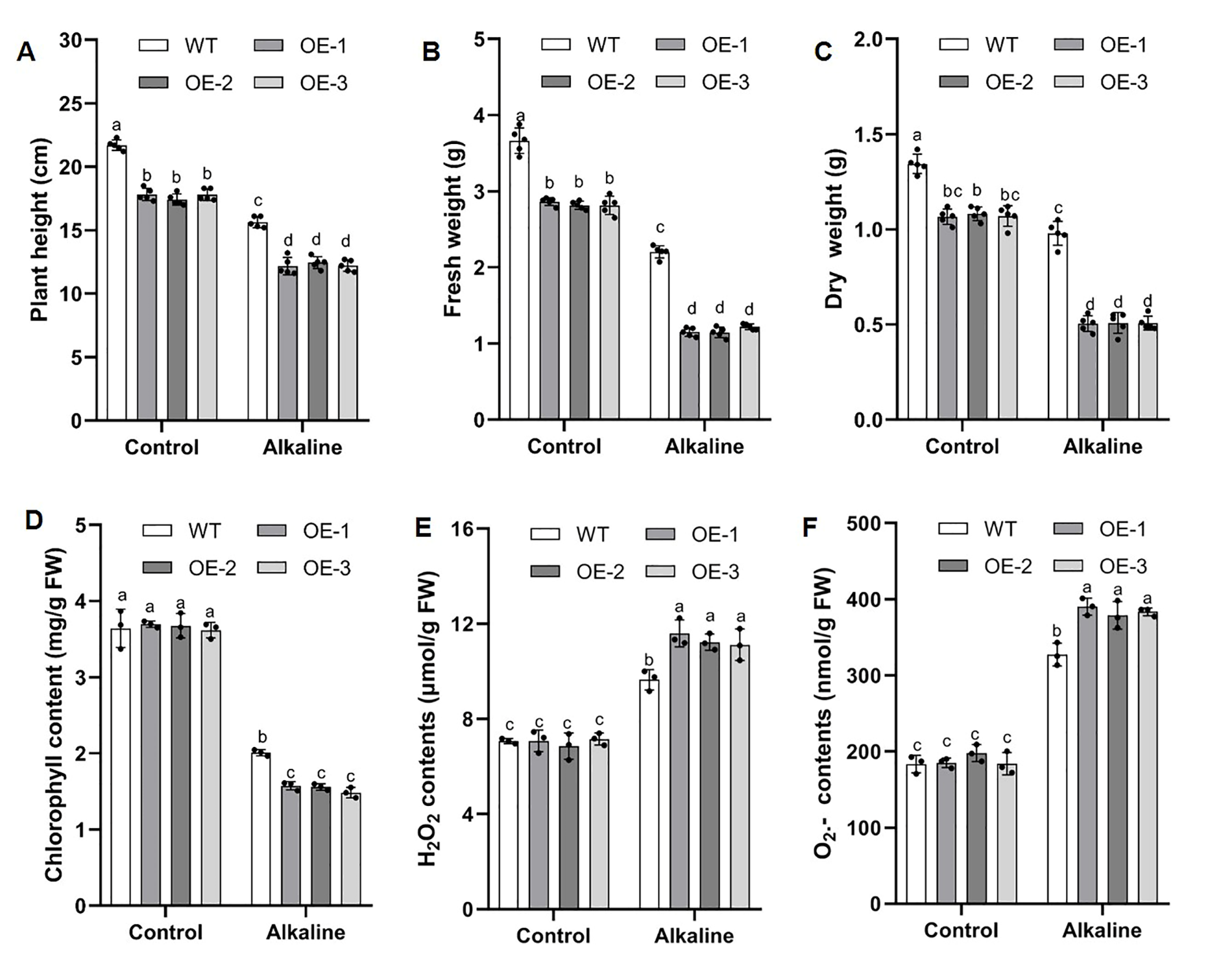


Figure S2. *MdNAC104* reduces alkaline resistance in apple. (A) Plant height, (B) Fresh weight, (C) Dry weight of transgenic *MdNAC104* apple. The data presented are mean ± standard deviation of five biological replicates. (D) Chlorophyll content. (E) H_2_O_2_ content. (F) O_2_^-.^ content. The data presented are means ± standard deviation of three biological replicates. Different letters indicate significant differences in values as determined by a one-way ANOVA Tukey’s test (*P* < 0.05).


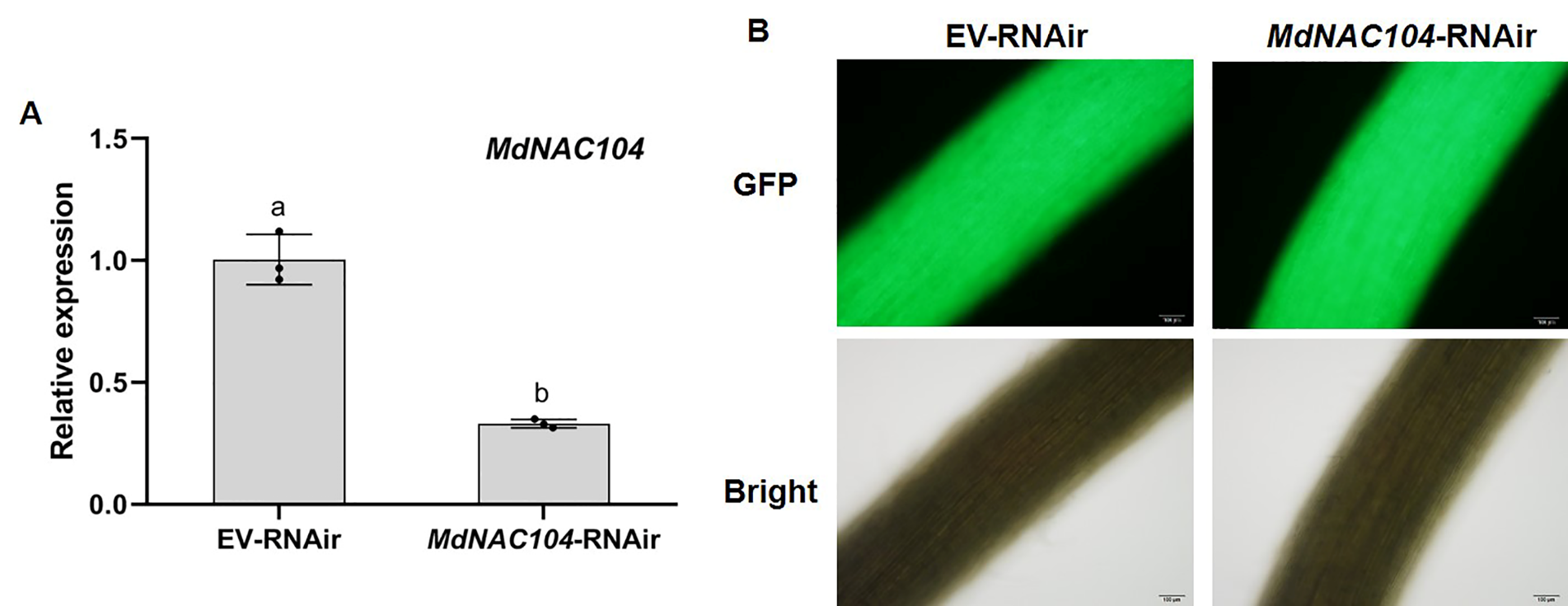
Figure S3. Identification of *MdNAC104* transgenic apple. (A) The relative expression of *MdNAC104* in EV-RNAir and MdNAC104-RNAir transgenic apple roots was detected by RT-qPCR. Data are mean ± standard deviation of three biological replicates. Student's *t*-test were used to determine statistical significance (*P* < 0.05). (B) Fluorescence identification of transformed plants from apple roots. Scale bar = 100 μm.


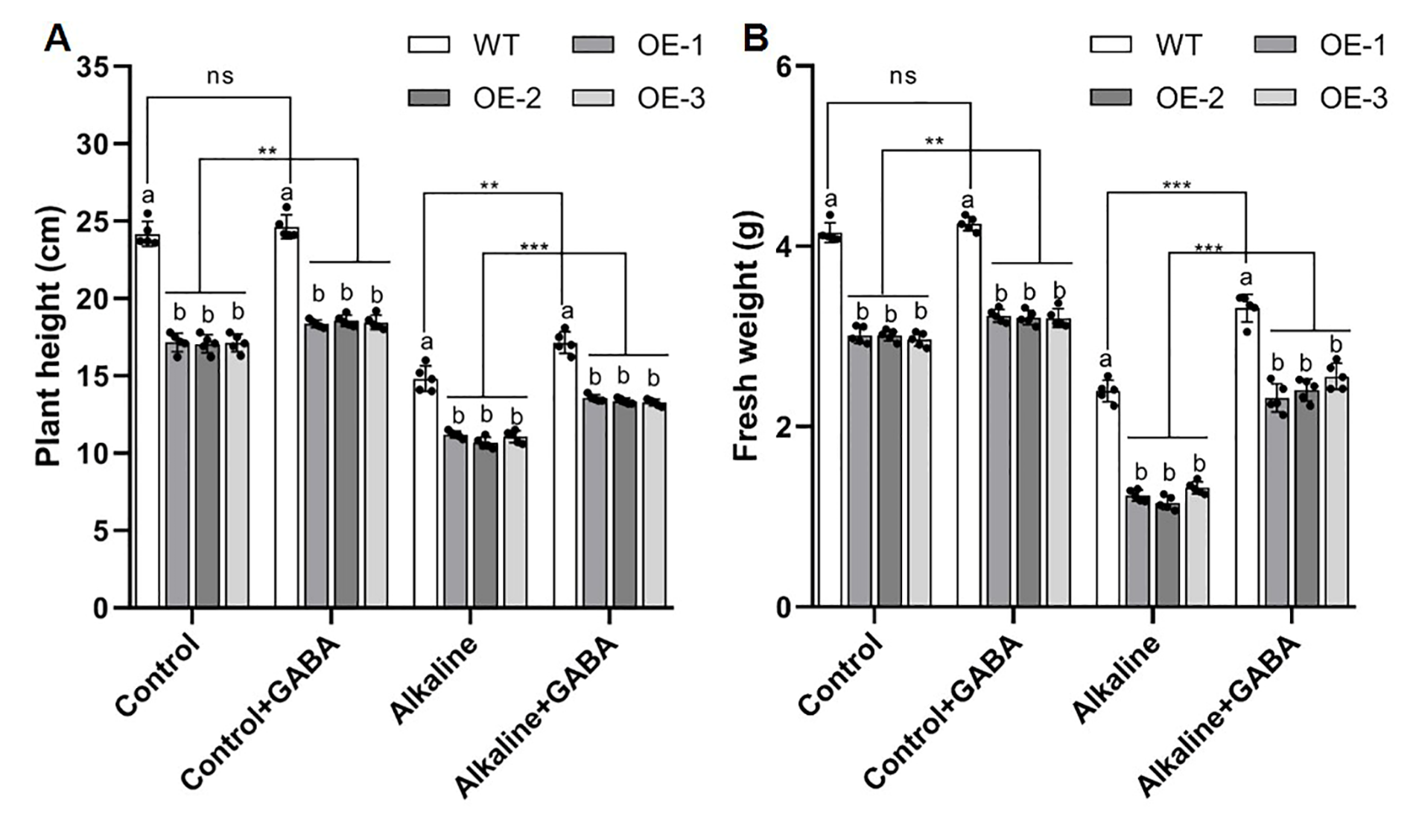
Figure S4. Exogenous GABA alleviated the phenotype of apple overexpressing *MdNAC104* under alkaline stress. (A) Plant height and (B) Fresh weight of transgenic *MdNAC104* apple. The data presented are mean ± standard deviation of five biological replicates. Data are mean ± standard deviation of five biological replicates. One-way ANOVA Tukey's test (*P* < 0.05) and Student's *t*-test were used to determine statistical significance (**P* < 0.05, ***P* < 0.01, ****P* < 0.001).


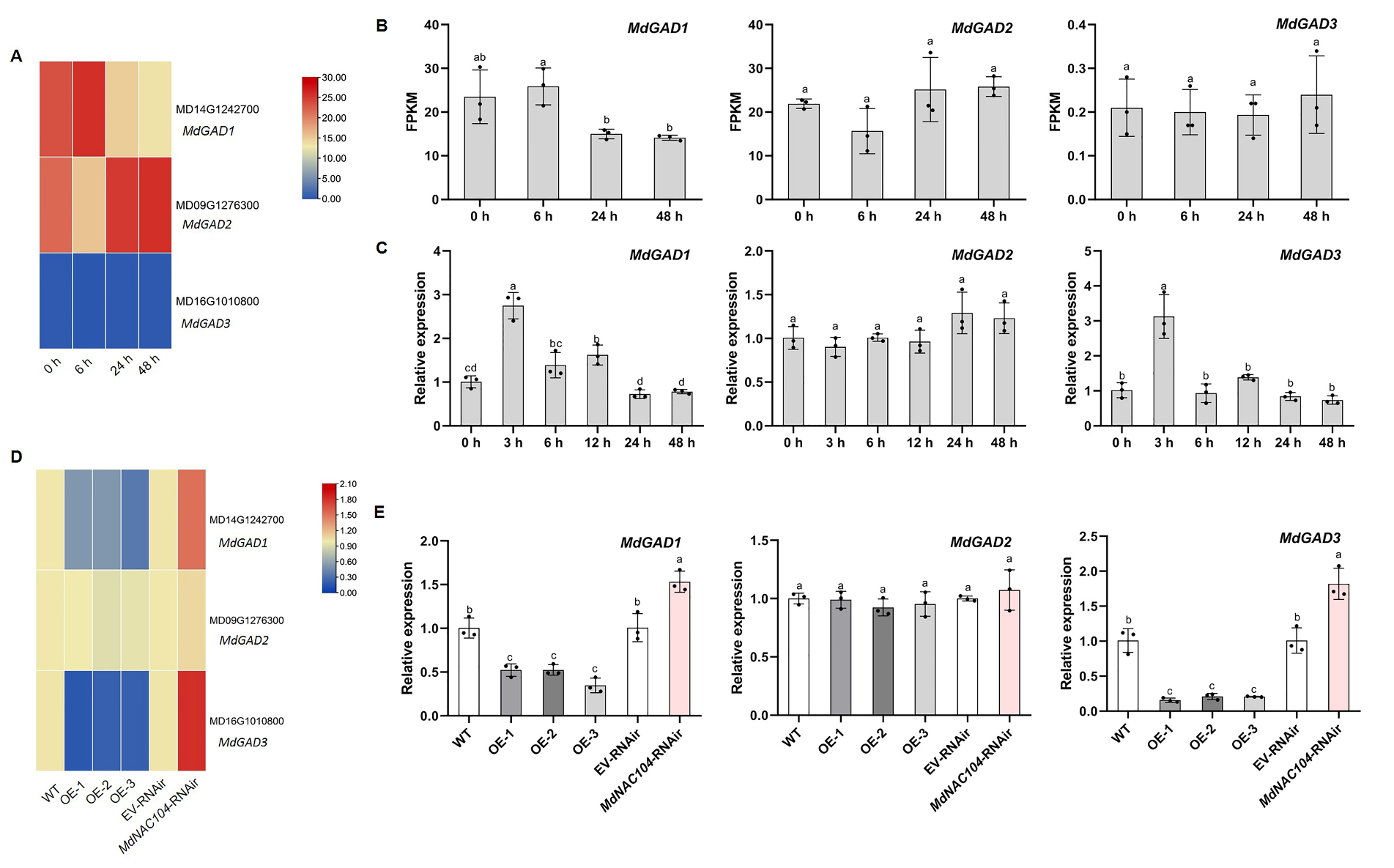
Figure S5. *MdGADs* expression levels in apple roots under alkaline stress. (A) Heatmap of the FPKM of *MdGADs* gene based on RNA-seq. (B) The FPKM of *MdGAD1, MdGAD2,* and *MdGAD3.* (C) RT-qPCR analysis of *MdGAD1* and *MdGAD3* expression under alkaline stress. (D) Heatmap of *MdGAD1*, *MdGAD2* and *MdGAD3* expression in *MdNAC104* transgenic apple, with the data in WT set as 1. (E) The expression of *MdGAD1*, *MdGAD2* and *MdGAD3* in *MdNAC104* transgenic apple. Data are mean ± standard deviation of three biological replicates. Different letters indicate significant differences in values as determined by a one-way ANOVA Tukey’s test (*P* < 0.05).


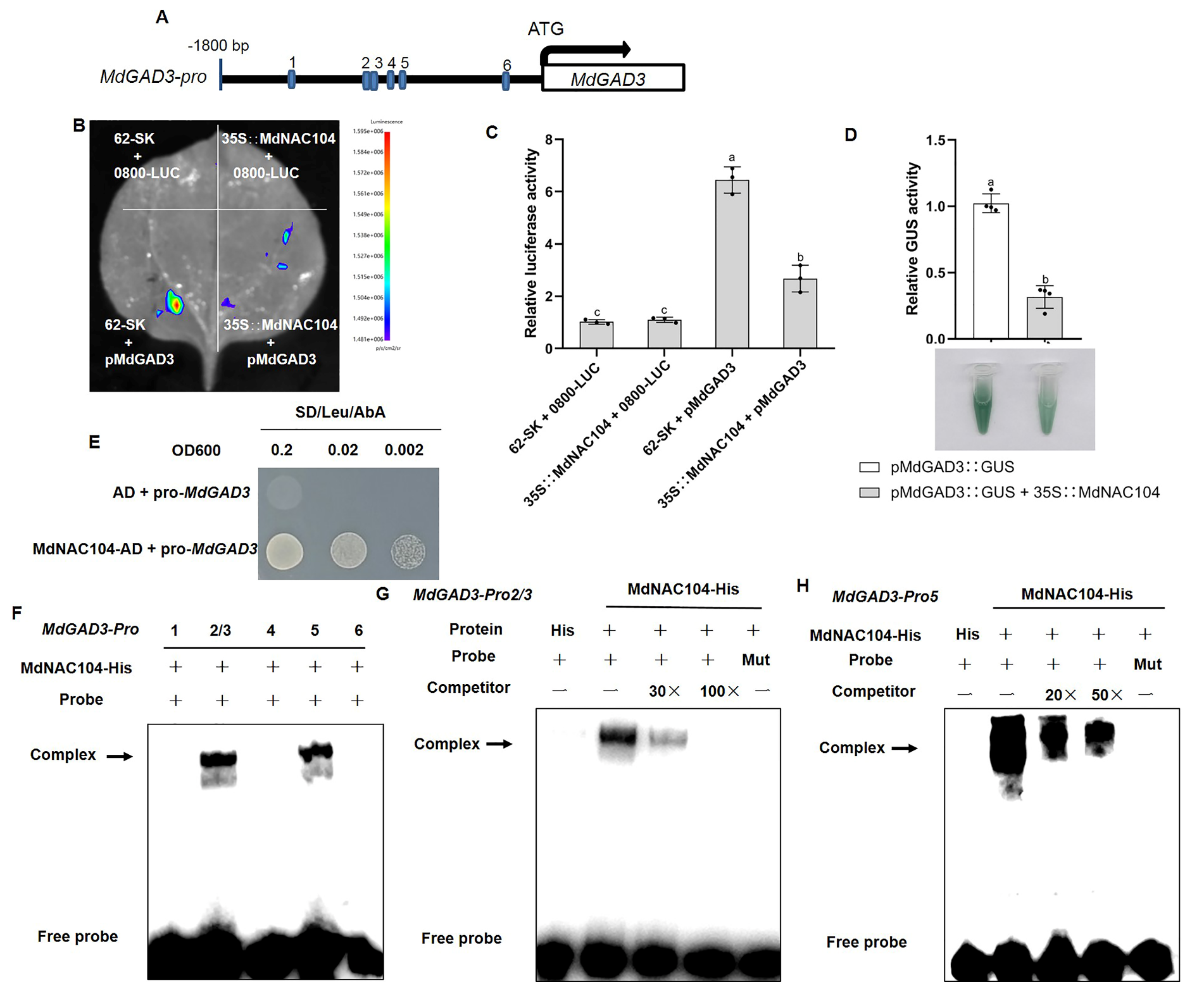
Figure S6. MdNAC104 inhibits the transcription of MdGAD3 and binds to its promoter. (A) ‘CACG’ elements in the upstream promoter region of *MdGAD3*. (B, C) Dual-luciferase assay showing that MdNAC104 could inhibit the expression of *MdGAD3*. The relative luciferase (LUC:REN) activity of 62-SK+0800-LUC was set to 1. The effector vector (35S::MdNAC104) and the luciferase (LUC) reporter vector (pMdGAD3::LUC). (D) Relative β-glucuronidase (GUS) activity. To observe the relationship between MdNAC104 and the MdGAD3 promoter, *Agrobacterium tumefaciens* containing pMdGAD3::GUS + EV and pMdGAD3::GUS + 35S::MdNAC104 were transformed into apple calli and stained. With pMdGAD3::GUS + EV as control, the activity of GUS was set as 1. Data are mean ± standard deviation of three biological replicates. One-way ANOVA Tukey's test and Student's *t*-test (*P* < 0.05) were used to determine statistical significance. (E) Y1H assay showing that MdNAC104 binds to the promoter regions of *MdGAD3* containing 'CACG'. Yeast cotransformed with the empty pGADT7 (AD) vector and the *MdGAD3* promoter was used as a negative control. Yeast cotransformed with pAbAi-MdGAD3 and MdNAC104-AD transformants grew well on media supplemented with 250 ng/mL AbA. (F-H) Identification of MdNAC104 binding specificity to sites 2/3 and 5 in *MdGAD3-Pro* by electrophoretic mobility shift assays. The '+' indicates the presence of relevant probes or proteins; '-' indicates the absence of relevant proteins; 'His' indicates pET-32a vectors (His tag); Mut indicates the mutant form of *MdGAD3-Pro* in which the 5′-CACG-3′ has been replaced by 5′-AAAA-3′. The arrow indicates the position of a protein-DNA complex after incubation of a biotin-labelled DNA probe with His-MdNAC104.


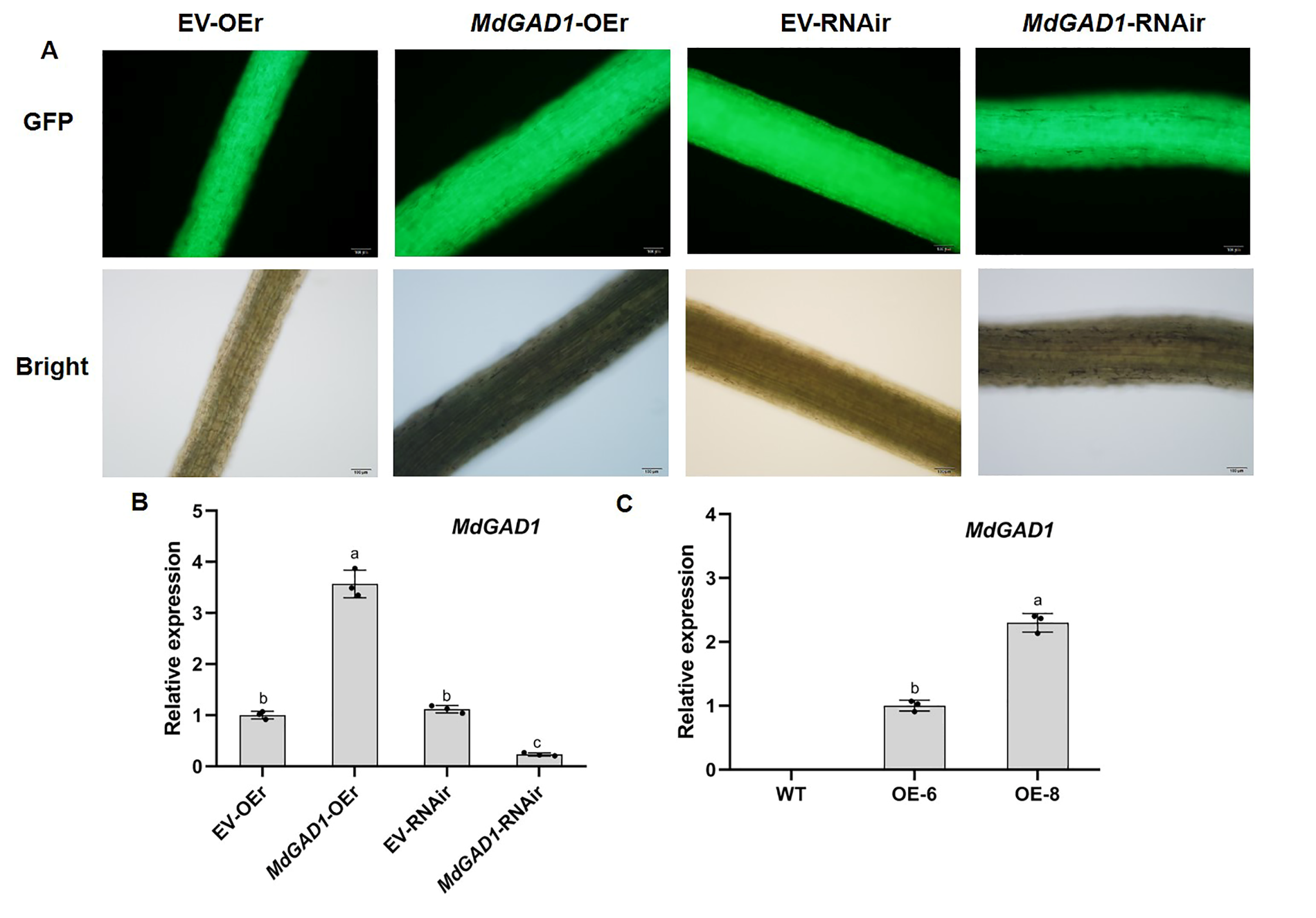
Figure S7. Identification of *MdGAD1* transgenic apple and tomato. (A) Fluorescence identification of transformed plants from apple roots. Scale bar = 100 μm. (B) The relative expression of *MdGAD1* in transgenic apple root and (C) tomato were detected by RT-qPCR. Data are means ± SD of 3 biological replicates. Different letters indicate significant differences in values as determined by a one-way ANOVA Tukey’s test or Student's *t*-test (*P* < 0.05).


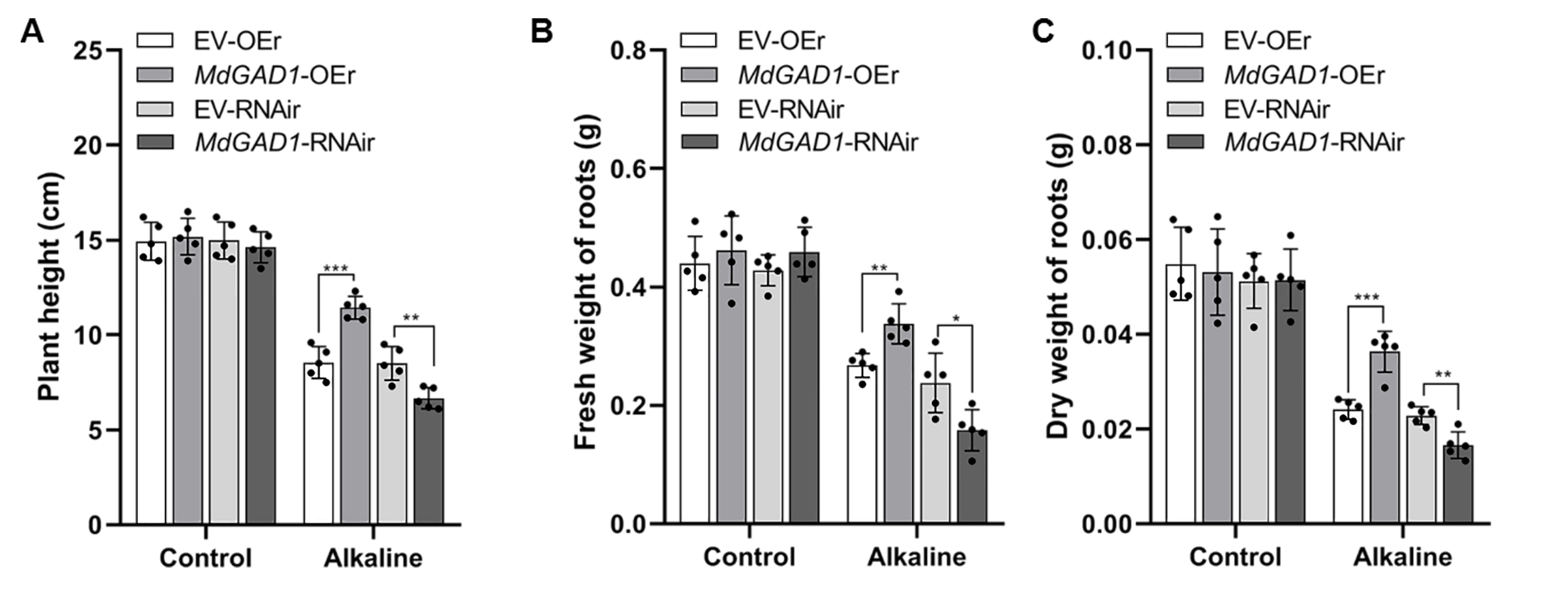
Figure S8. MdGAD1 positively regulates alkaline resistance in apple. (A) Plant height, (B) Fresh weight of roots, (C) Dry weight of roots of transgenic *MdGAD1* apple roots. The data presented are mean ± standard deviation of five biological replicates. Student's *t*-test was used to determine statistical significance (**P* < 0.05, ***P* < 0.01; ****P* < 0.001).


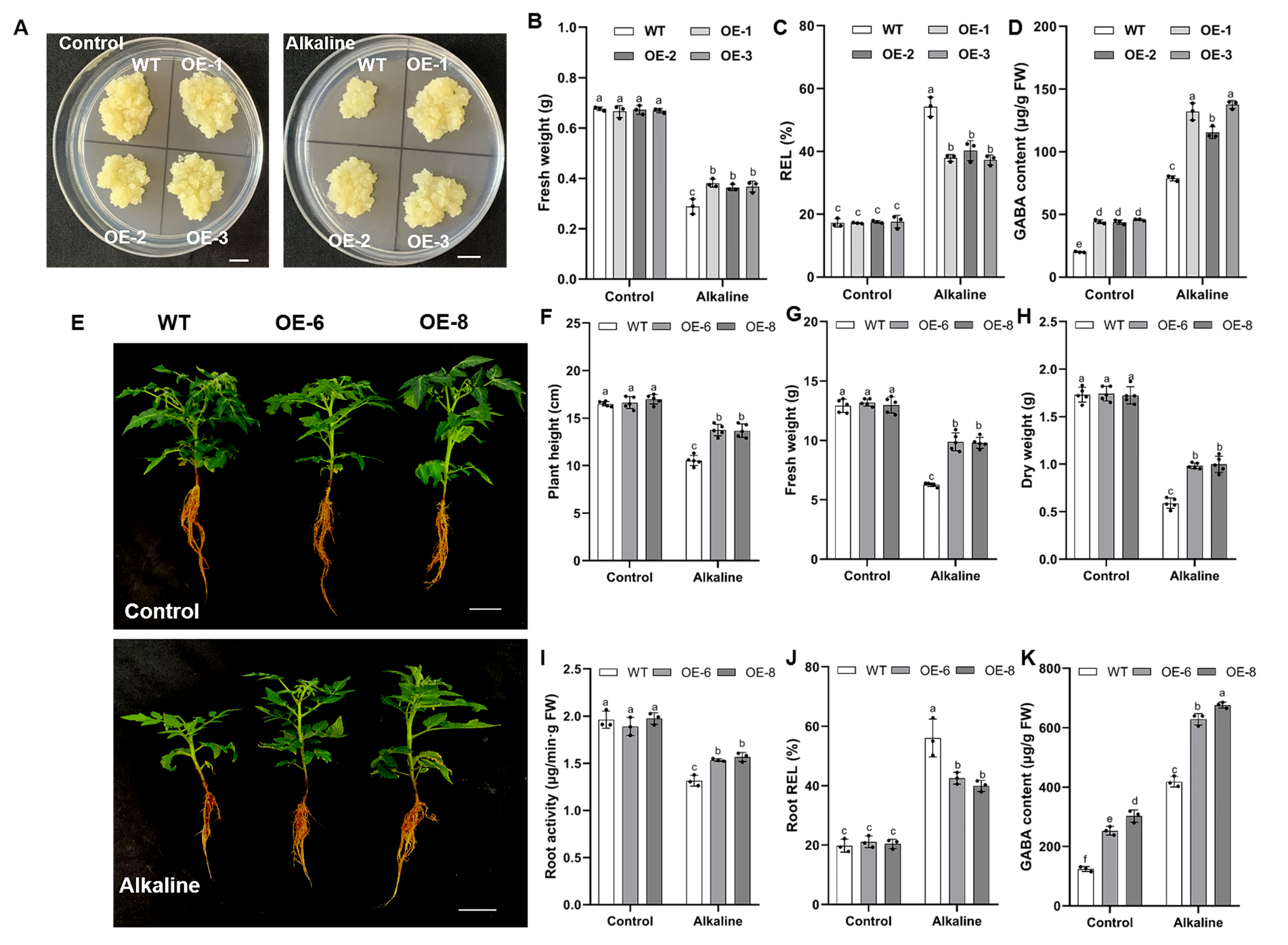
Figure S9. Overexpression of *MdGAD1* improves alkaline resistance in transgenic calli and tomatoes. (A) Growth phenotypes, (B) Fresh weight, (C) Relative electrolyte leakage (REL) and (D) GABA content of *MdGAD1* transgenic calli under alkaline treatment for 18 d. Bars = 1 cm. (E) Heterologous overexpression of *MdGAD1* phenotype under alkaline stress (NaHCO_3_: Na_2_CO_3_ = 1:1). Bars = 5 cm. (F) Plant height, (G) Fresh weight, and (H) Dry weight. Data are mean ± standard deviation of five biological replicates. (I) Root activity, (J) Root relative electrolyte leakage (Root REL) and (K) GABA content of transgenic *MdGAD1* tomatoes. Data are mean ± standard deviation of three biological replicates. Different letters indicate significant differences in values as determined by a one-way ANOVA Tukey’s test (*P* < 0.05).


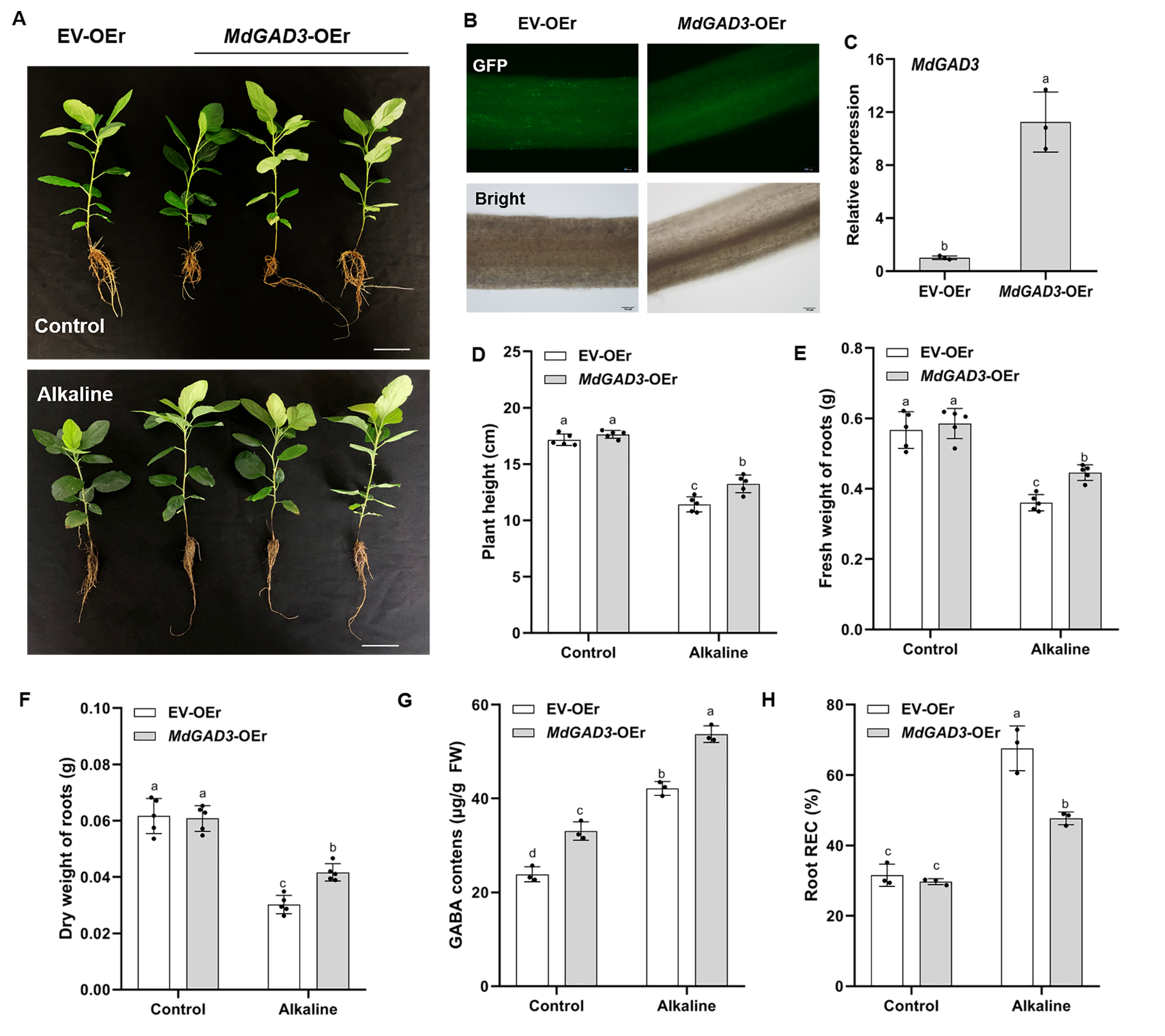


Figure S10. Overexpression of *MdGAD3* improves alkaline resistance in apples. (A) Phenotypes of *MdGAD3*-OEr (*MdGAD3*-OE roots) treated for 15 d under hydroponic culture for alkaline stress (NaHCO_3_: Na_2_CO_3_ = 1:1), Bars = 5 cm. (B, C) Identification of *MdGAD3* transgenic apple. (D) Plant height, (E) Fresh weight of roots, (F) Dry weight of roots, (G) Root activity and (H) Root relative electrolyte leakage (Root REL) of transgenic *MdGAD3* apple. Data are mean ± standard deviation of five biological replicates (D-F) or three biological replicates (C, G-H). Different letters indicate significant differences in values as determined by a one-way ANOVA Tukey’s test or Student's *t*-test (*P* < 0.05).


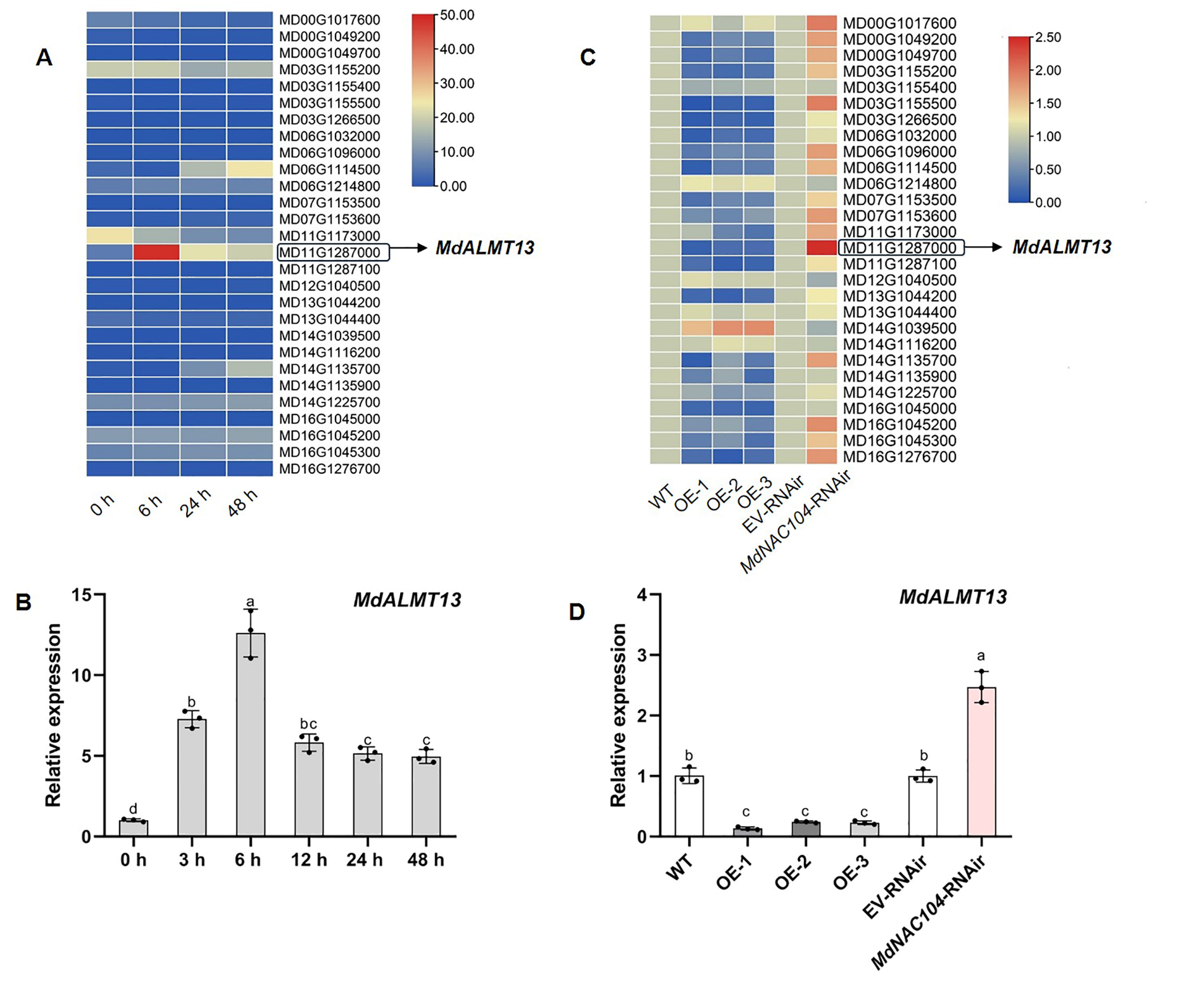
Figure S11. *MdALMTs* expression levels in apple roots under alkaline stress. (A) Heatmap of the FPKM of *MdALMTs* gene based on RNA-seq. (B) RT-qPCR analysis of *MdALMT13* expression under alkaline stress. (C) Heatmap of *MdALMTs* expression in *MdNAC104* transgenic apple, with the data in WT set as 1. (D) The expression of *MdALMT13* in *MdNAC104* transgenic apple. Data are mean ± standard deviation of three biological replicates. Different letters indicate significant differences in values as determined by a one-way ANOVA Tukey’s test (*P* < 0.05).


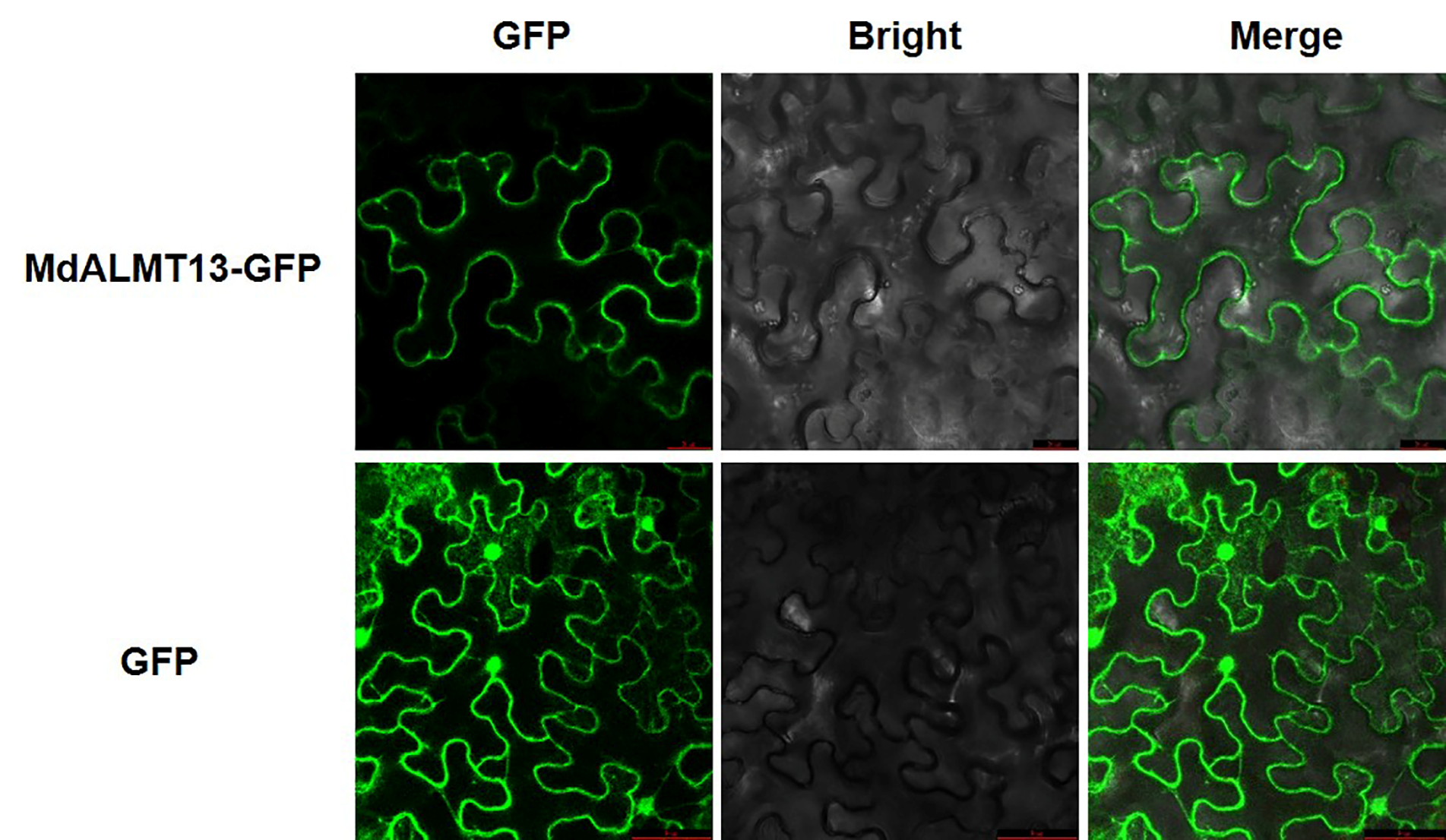


Figure S12. Subcellular localization analysis of MdALMT13-GFP fusion protein in *Nicotiana benthamiana* epidermal cells. MdALMT13-GFP Scale bar = 20 μm. GFP Scale bar = 50 μm.


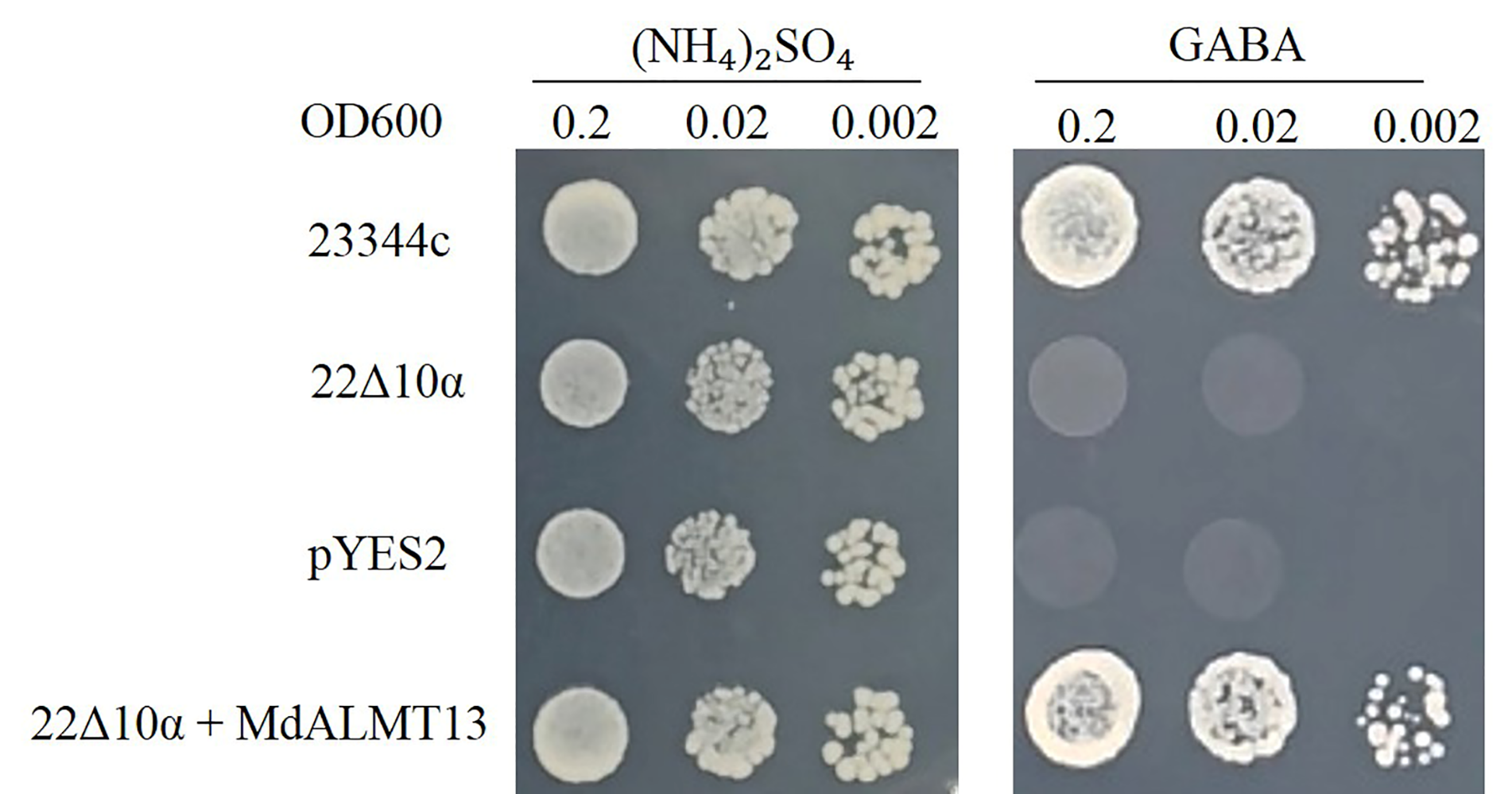


Figure S13. The amino acid transport deficient yeast strain 22Δ10α and wild type yeast strain 23344c were used as the research object to analyse the growth of yeast cells expressing the MdALMT13 gene on non-selective solid medium containing 1 mM (NH_4_)_2_SO_4_ or selective culture with 1 mM GABA as the sole nitrogen source. The *MdALMT13* gene was cloned into pYES2 and then transferred into 22Δ10α. Wild type yeast strain 23344c was used as positive control. 22Δ10α and pYES2 were used as negative control. Photographs were taken after 48 hours of growth at 30°C.


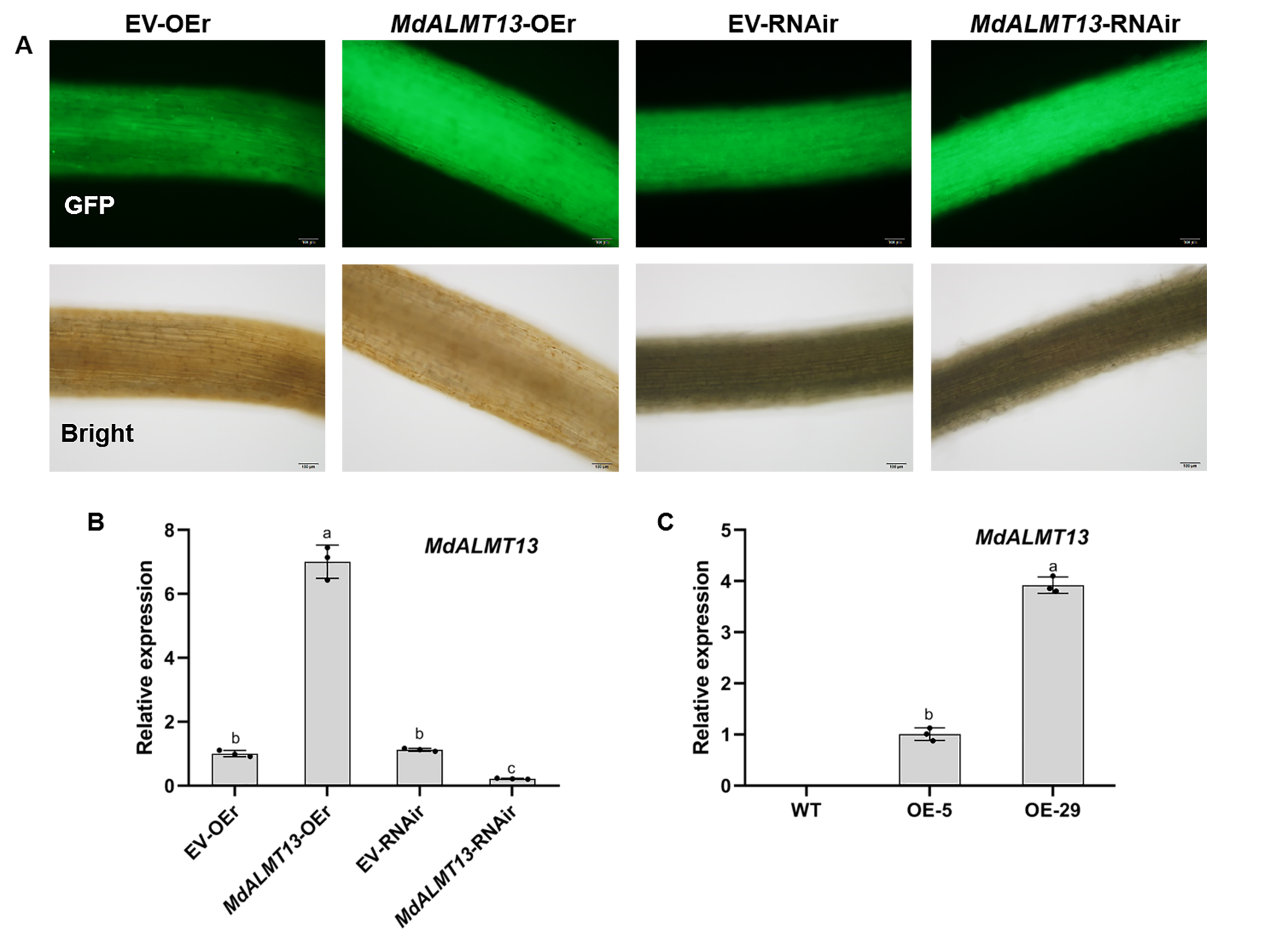
 Figure S14. Identification of *MdALMT13* transgenic apple and tomato. (A) Fluorescence identification of transformed plants from apple roots. Scale bar = 100 μm. (B) The relative expression of *MdALMT13* in transgenic apple root and (C) tomato were detected by RT-qPCR. Data are mean ± standard deviation of three biological replicates. Different letters indicate significant differences in values as determined by a one-way ANOVA Tukey’s test or Student's *t*-test (*P* < 0.05).


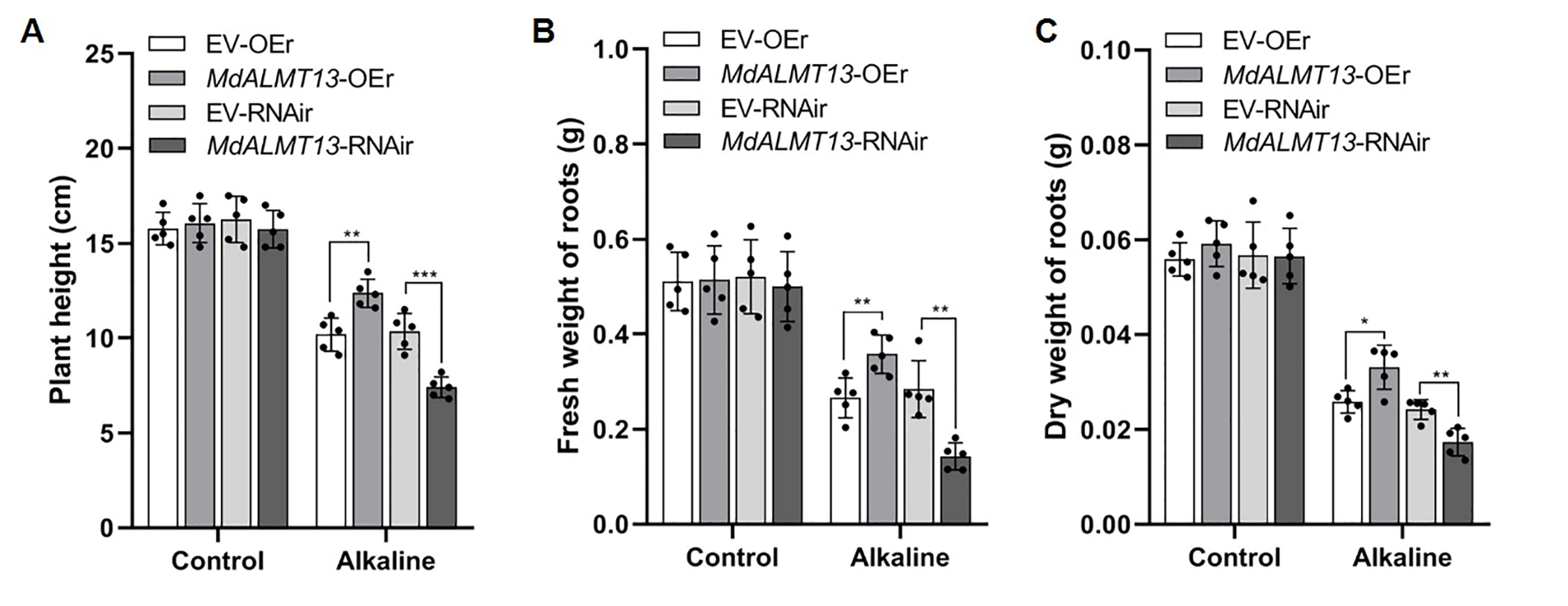
 Figure S15. MdALMT13 positively regulates alkaline resistance in apple. (A) Plant height, (B) Fresh weight of roots, (C) Dry weight of roots of transgenic *MdALMT13* apple roots. The data presented are mean ± standard deviation of five biological replicates. Student's *t*-test was used to determine statistical significance (**P* < 0.05; ***P* < 0.01; ****P* < 0.001).


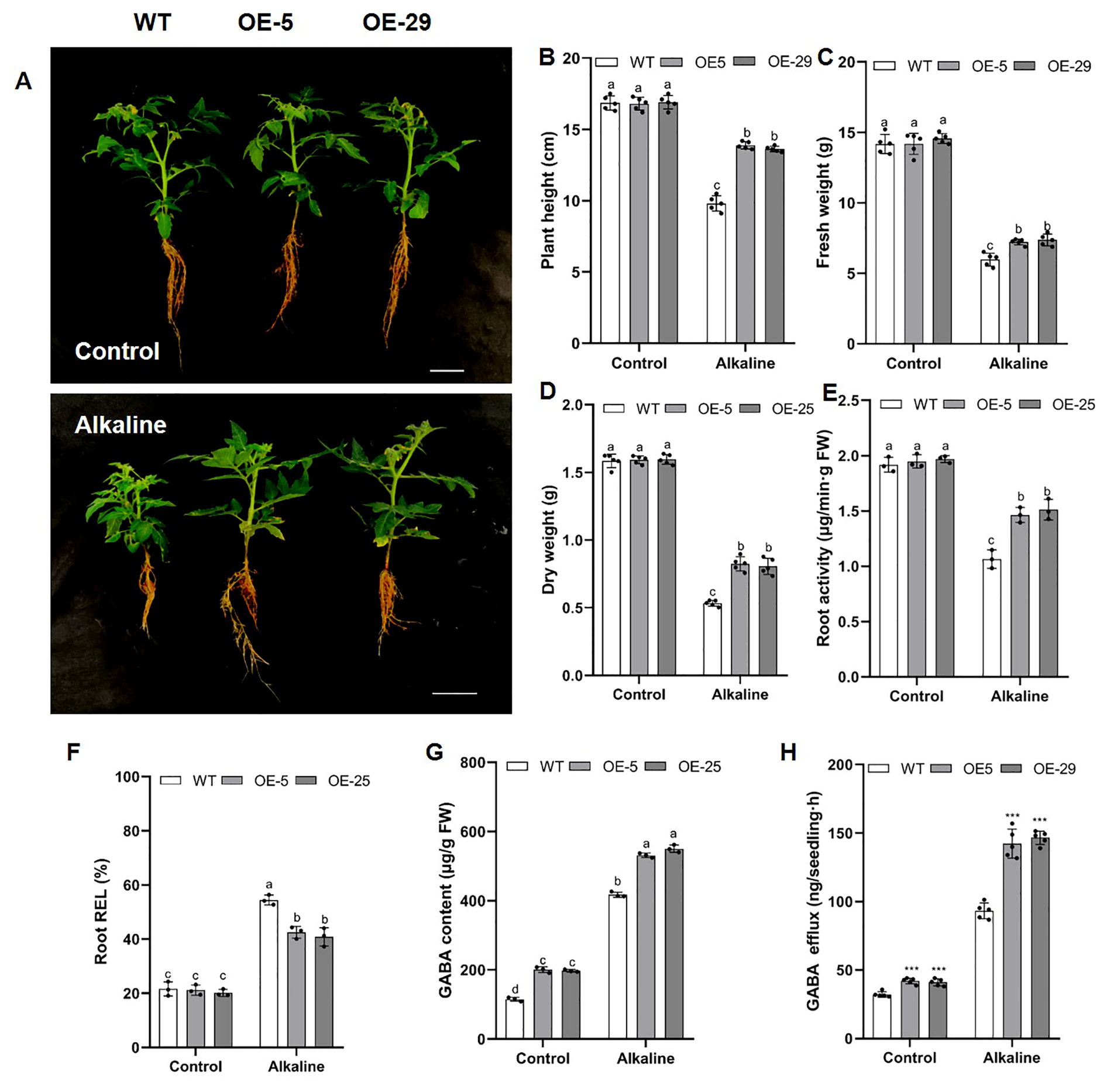
 Figure S16. Heterologous overexpression of *MdALMT13* improves alkaline resistance in transgenic tomato. (A) Heterologous overexpression of *MdALMT13* phenotype under alkaline stress (NaHCO_3_: Na_2_CO_3_ = 1:1). Bars = 5 cm. (B) Plant height, (C) Fresh weight, and (D) Dry weight. Data are mean ± standard deviation of five biological replicates. (E) Root activity, (F) Root relative electrolyte leakage (Root REL) and (G) GABA content of transgenic MdALMT13 tomatoes. Data are mean ± standard deviation of three biological replicates. Different letters indicate significant differences in values as determined by a one-way ANOVA Tukey’s test (*P* < 0.05). (H) Net GABA efflux. Data are mean ± standard deviation of five biological replicates. Student's *t*-test were used to determine statistical significance (**P* < 0.05, ***P* < 0.01, ****P* < 0.001).


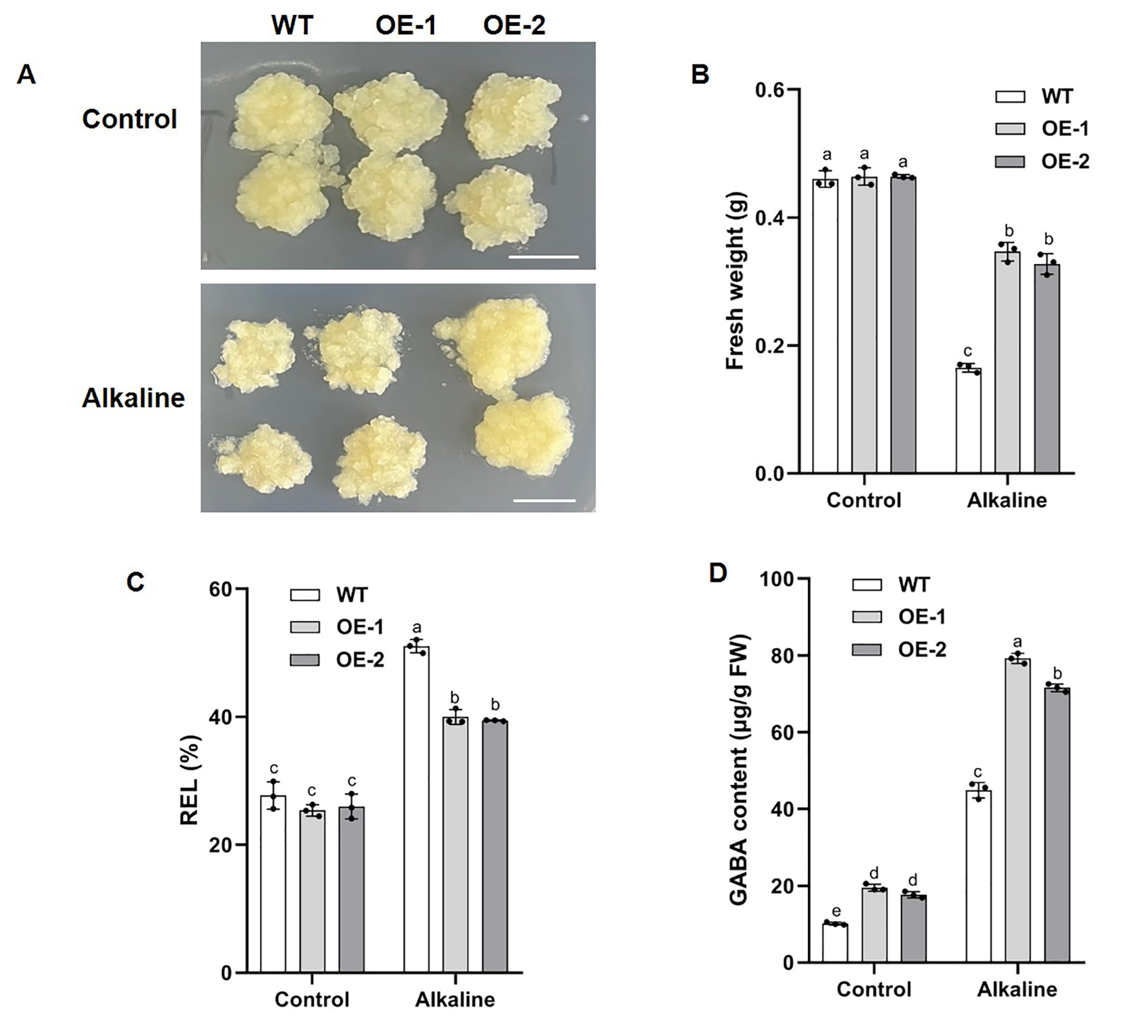
 Figure S17. MdALMT13 improved alkaline resistance in apples calli. (A) Phenotype of *MdALMT13* transgenic calli under alkaline stress for 15 d. Bars = 1 cm. (B) fresh weight, (C) REL and (D) GABA content in transgenic *MdALMT13* apple calli. Data are mean ± standard deviation of three biological replicates. Different letters indicate significant differences in values as determined by a one-way ANOVA Tukey’s test (*P* < 0.05).


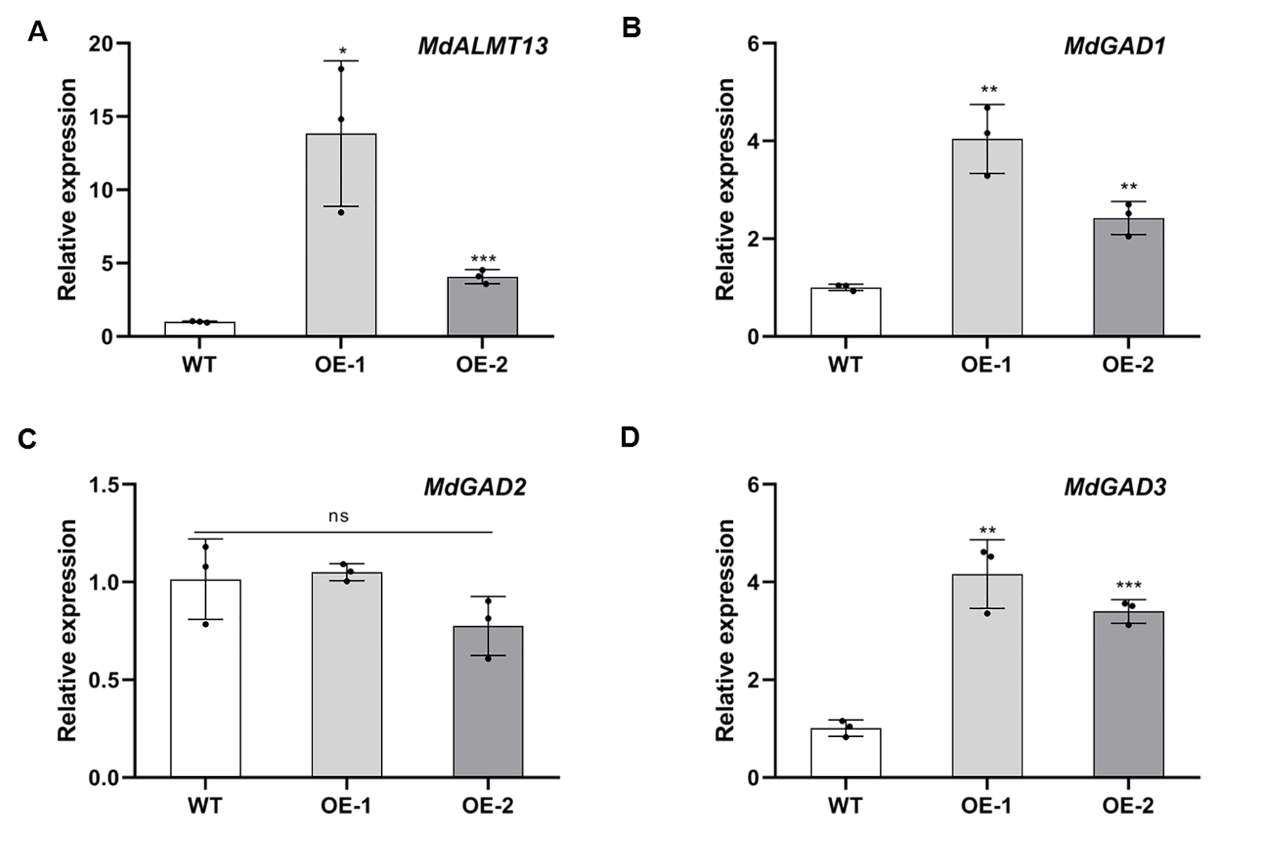
 Figure S18. The expression of *MdALMT13*, *MdGAD1*, *MdGAD2* and *MdGAD3* in *MdALMT13* transgenic apple calli. Data are mean ± standard deviation of three biological replicates. Student's *t*-test was used to determine statistical significance (**P* < 0.05, ***P* < 0.01, ****P* < 0.001).


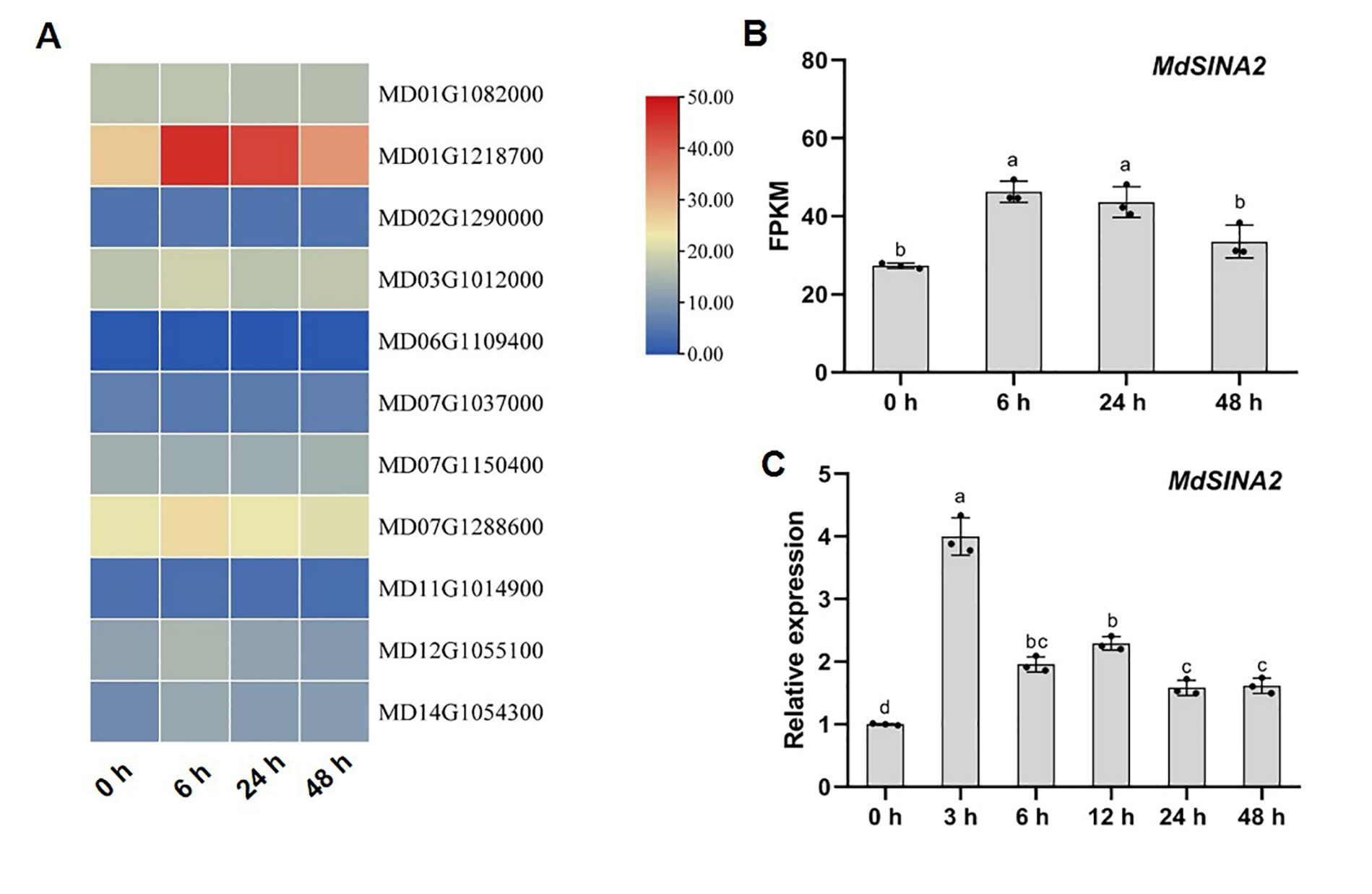
 Figure S19. *MdSINAs* expression levels in apple roots under alkaline stress. (A) Heatmap of FPKM of *MdSINAs* gene based on RNA-seq. (B) The FPKM of *MdSINA2.* (C) RT–qPCR analysis of *MdSINA2* expression under alkaline stress. Data are mean ± standard deviation of three biological replicates. Different letters indicate significant differences in values as determined by a one-way ANOVA Tukey’s test (*P* < 0.05).


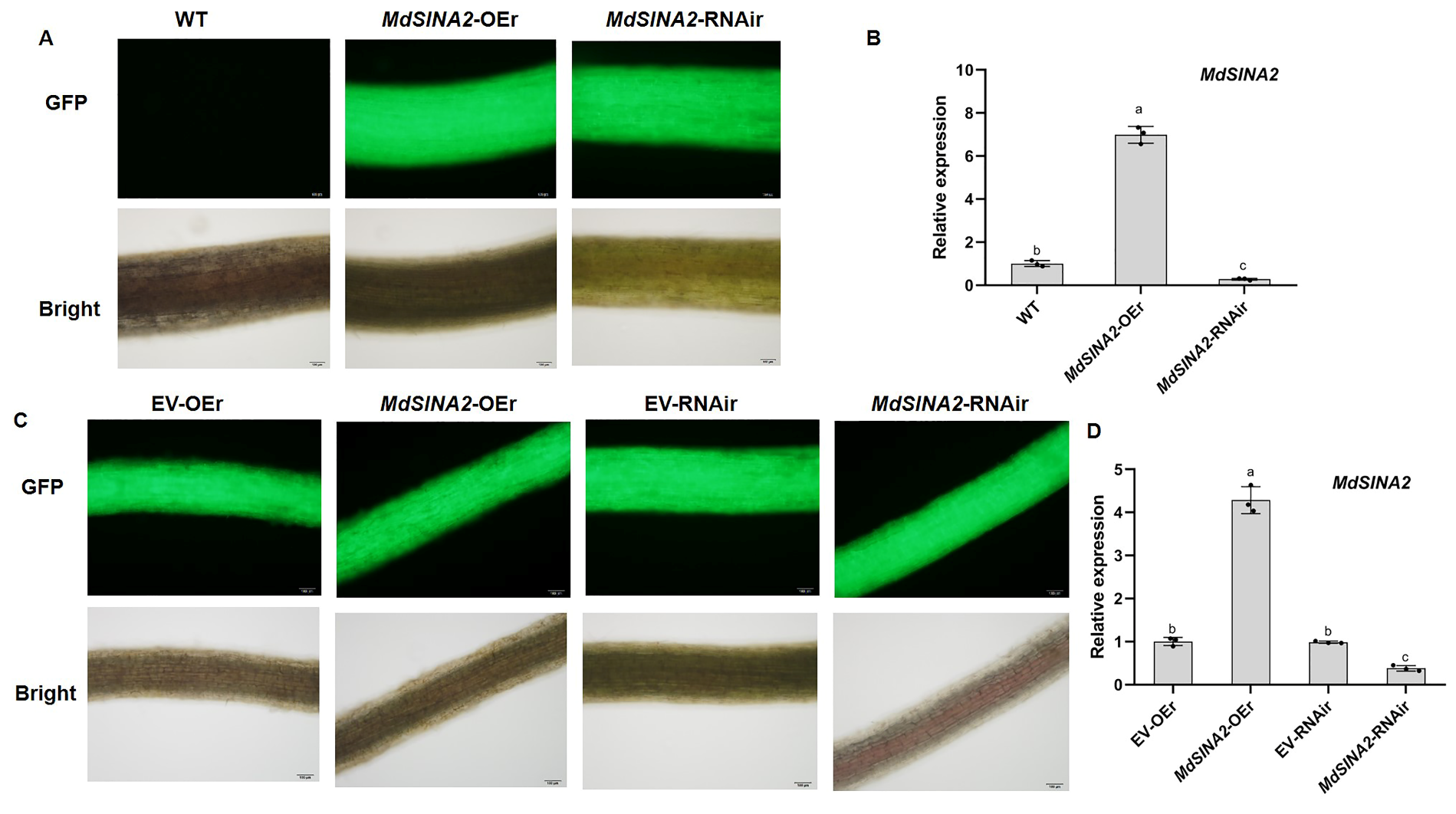
 Figure S20. Identification of *MdSINA2* transgenic apple. (A, C) Fluorescence identification of transformed plants from apple roots. Scale bar = 100 μm. (B, D) The relative expression of *MdSINA2* in transgenic apple root was detected by RT-qPCR. Data are mean ± standard deviation of three biological replicates. Different letters indicate significant differences in values as determined by a one-way ANOVA Tukey’s test (*P* < 0.05).


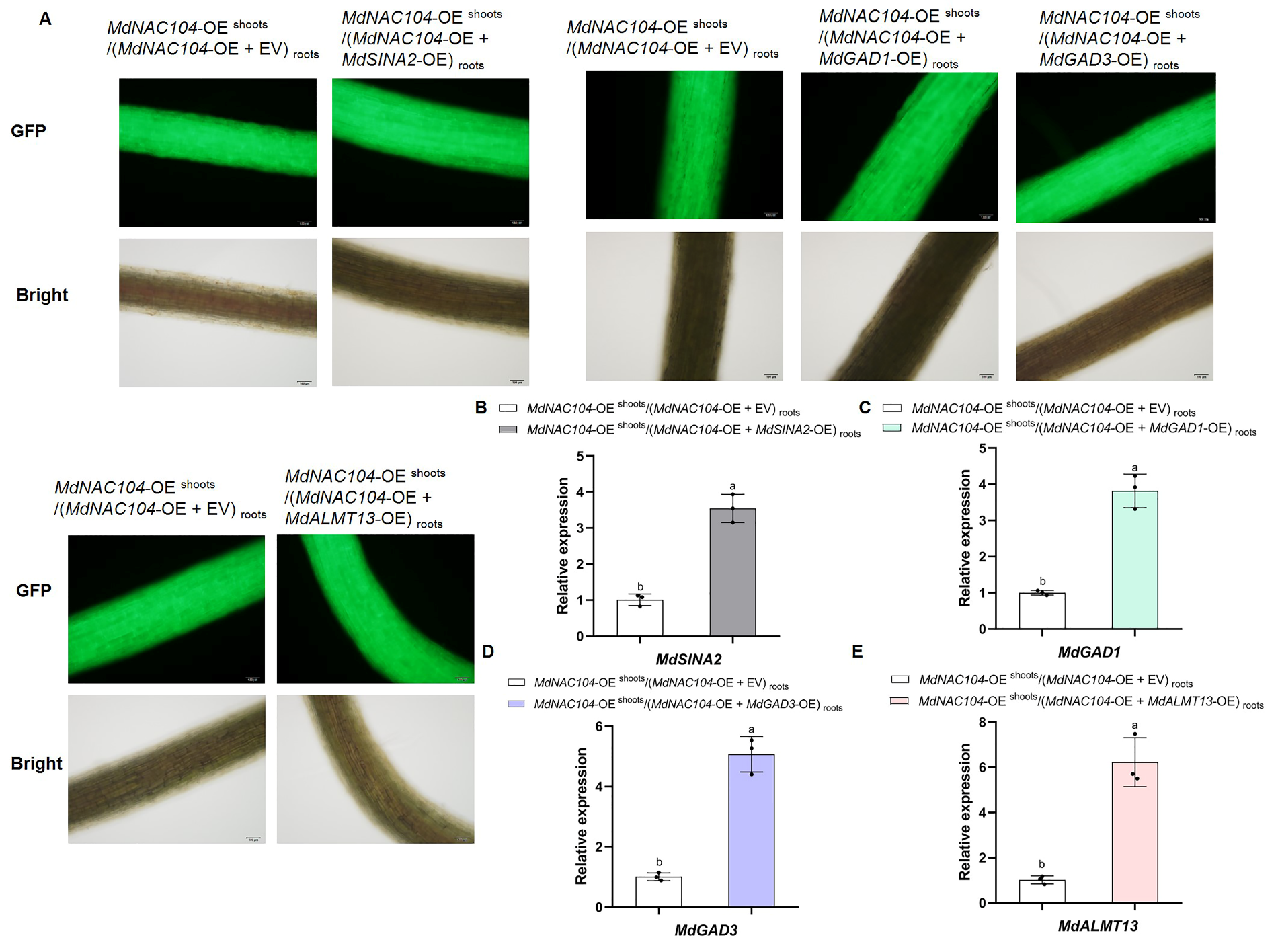
Figure S21. (A) Fluorescence identification of transformed plants from apple roots. Scale bar = 100 μm. (B) RT-qPCR analysis of expression of *MdSINA2*, *MdGAD1*, *MdGAD3*, and *MdALMT13* in roots of chimeric plants with roots carrying the *MdNAC104*-OE ^shoots^ /(*MdNAC104*-OE + EV) _roots_, *MdNAC104*-OE ^shoots^ /(*MdNAC104*-OE + *MdSINA2*-OE) _roots_, *MdNAC104*-OE ^shoots^ /(*MdNAC104*-OE + *MdGAD1*-OE) _roots_, *MdNAC104*-OE ^shoots^ /(*MdNAC104*-OE + *MdGAD3*-OE) _roots_, or *MdNAC104*-OE ^shoots^ /(*MdNAC104*-OE + *MdALMT13*-OE) _roots_ constructs. Data are mean ± standard deviation of three biological replicates. Student's *t*-test was used to determine statistical significance (*P* < 0.05).
